# Mast cells initiate lymphocyte egress from distant lymph nodes upon skin inflammation via RANKL/sphingosine-1-phosphate axis

**DOI:** 10.1172/JCI199237

**Published:** 2026-09-24

**Authors:** Konstantinos Katsoulis-Dimitriou, Waqar Umer, Laura Knop, Tanja Schickschneit, Aaron Hoffmann, Ali El-Bizri, Lea M. Schmitter, Kathleen Baumgart, Nouria Jantz-Naeem, Vladyslava Dovhan, Charlotte Heidelbach, Lars Philipsen, Andreas J Müller, Stephan Fricke, Sascha Kahlfuß, Thomas Schüler, Jan Dudeck, Anne Dudeck

**Affiliations:** 1 Institute for Clinical Immunology and Cell Therapeutics, Medical Faculty,; 2 Multiparametric Bioimaging and Cytometry Platform, Medical Faculty, and; 3 Health Campus Immunology, Infectiology and Inflammation (G-CI3), Otto-von-Guericke-Universität Magdeburg, Magdeburg, Germany.

**Keywords:** Dermatology, Immunology, Inflammation, Adaptive immunity, Mast cells, T cells

## Abstract

Receptor activator of NF-κB ligand (RANKL) is important for bone metabolism, but also modulates immune processes. We showed that mast cells (MCs) were involved in RANKL regulation, but the importance of MC-derived RANKL in skin inflammation has not, to our knowledge, been investigated. In contact hypersensitivity (CHS), the absence of MC-derived RANKL led to reduced skin inflammation due to impaired leukocyte infiltration and blood lymphopenia. Surprisingly, we observed a massive hyperplasia of the distant inguinal lymph nodes (LNs) in the absence of MC RANKL. Using adoptive transfers, flow cytometry, and whole-mount 3D imaging, we demonstrated that this was not caused by structural maladaptation, but rather by the inability of lymphocytes to exit in a timely manner. Importantly, RANKL deletion in skin MCs only replicated the effect of LN hyperplasia and blood lymphopenia. Moreover, MCs were involved in serum sphingosine-1-phosphate (S1P) regulation during sensitization and challenge. Intravascular administration of S1P restored timely lymphocyte egress, demonstrating a MC-induced, organ-spanning RANKL/S1P axis. Consequently, peripheral skin MC–derived RANKL was essential for the timely lymphocyte egress from distant LNs, which may have important implications for the targeted treatment of inflammatory skin diseases.

## Abstract 2

<strong>Katsoulis-Dimitriou et al. showed that, during skin inflammation, peripheral mast cells signal remotely to distant lymph nodes and initiate lymphocyte egress via a RANKL-sphingosine-1-phosphate axis.</strong>

## Graphical abstract

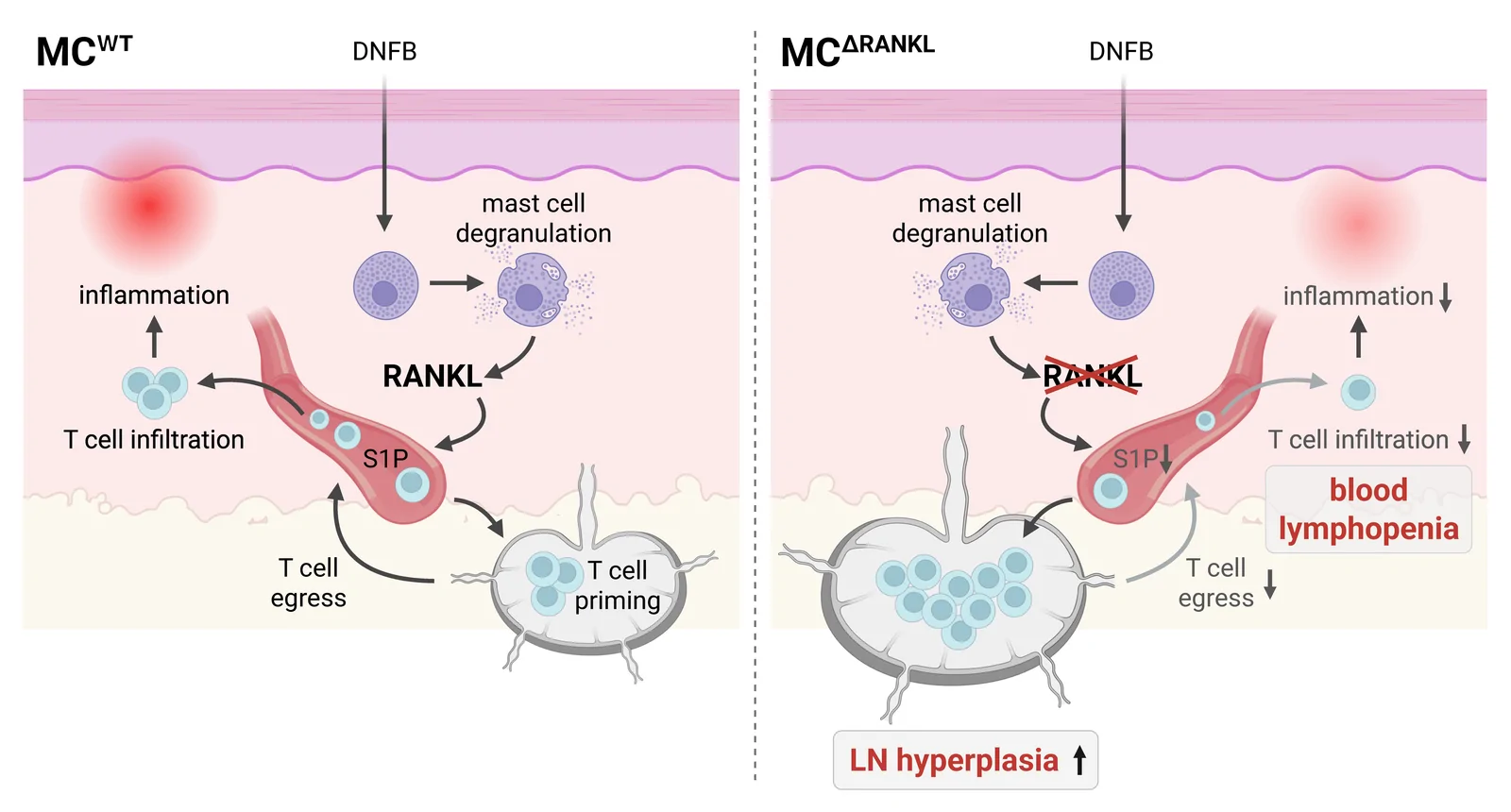

## Introduction

Receptor activator of NF-κB ligand (RANKL) and its receptor RANK are members of the TNF superfamily and best known for their role in skeletal development and bone homeostasis. RANKL stimulates osteoclastogenesis, and thus bone resorption, and thereby also promotes bone loss during age-related osteoporosis and inflammatory diseases (1, 2). However, there is increasing evidence for immune-regulating RANKL functions. As reviewed by Mueller and Hess, RANKL is involved in lymph node (LN) formation, since mice lacking RANKL or RANK expression completely lack LNs and show abnormalities in secondary lymphoid organs (SLOs) (3). In detail, RANKL released by lymphoid tissue–organizer (LTO) cells is responsible for the recruitment of lymphoid tissue–inducer (LTI) cells, which is crucial for LN formation (3). Moreover, RANKL initiates SLO development and promotes the growth of LNs, Peyer’s patches (PPs), and other SLOs (4, 5). Of note, RANKL has also been shown to modulate skin inflammation. Upon UV irradiation, RANKL released by keratinocytes interacts with RANK on Langerhans cells and dermal DCs and promotes T cell activation and Treg expansion (6, 7).

Mast cells (MCs) are long-lived, tissue-resident innate immune cells populating predominantly interface tissues, such as the skin. Given their immediate response to IgE/FcεRI crosslinking, MCs are best known as key effector cells in type I allergic reactions. MCs contain a huge amount of secretory granules that are filled with preformed mediators and instantaneously released by degranulation, which is later followed by the release of de novo–synthesized mediators (8). However, MCs also sense pathogen patterns, inflammatory stimuli, and cell stress signals, such as IL-33 and extracellular ATP (9, 10), and also play important roles in infections and (nonallergic) inflammatory diseases (11). Moreover, MCs drive the innate immune response and modulate adaptive immunity by direct communication with other immune cells (12). For example, we demonstrated a bidirectional interaction between MCs and DCs, in which DCs transfer MHC II complexes to MCs (13), while in turn engulfing intact MC granules, which promotes DC maturation, migration to draining LNs (dLNs), and their T cell–priming efficiency (14). Recently, we proved that perivascular skin MCs form intravascular sheets and degranulate directionally in the bloodstream. Preformed TNF, embedded in MC granules and infused via this route into the bloodstream, was crucial for priming blood circulating neutrophils to license their migration to the inflamed skin (15).

We have shown that MCs are involved in the increase of serum RANKL levels upon bone fracture healing and promote osteoclast formation (16). Using an MC-specific conditional KO, we have also demonstrated that MC-derived RANKL is involved in ovariectomy-induced osteoporosis (17). Considering the high density and sentinel function of MCs in the skin, we examined here the role of MC-derived RANKL in skin inflammation. We found that the release of RANKL by skin MCs was crucial for skin inflammation, but, surprisingly, this occurred by initiating lymphocyte egress from distant inguinal LNs, which is mandatory for their migration into the skin and proinflammatory effects.

## Results

We generated a conditional RANKL-KO mouse line by crossing RANKL^fl/fl^ and Mcpt5-Cre mice (17, 18), in which Cre expression was specific for connective tissue-type MCs. Efficient abrogation of RANKL expression has been confirmed in vivo in ear skin MCs and in vitro in peritoneal cultured MCs of Mcpt5-Cre^+^ RANKL^fl/fl^ (MC^ΔRANKL^) mice as compared with Cre^-^ control mice (MC^WT^) (Supplemental Figure 1A; supplemental material available online with this article; https://doi.org/10.1172/JCI199237DS1). (17). Proof of KO was possible only with specific antibodies (see Methods). Importantly, both MC numbers and distance to blood vessels were not altered upon RANKL deletion (Supplemental Figure 1B). MC ability to degranulate upon stimulation with calcium ionophore, ATP, ATP, and IL-33, or crosslinking of the IgE receptor was also unaltered (Supplemental Figure 1C). Given the relevance of RANKL in lymphoid tissue development, we analyzed LN and blood immune cell populations in steady state. Through immunophenotyping, we observed no difference between MC^ΔRANKL^ and MC^WT^ in LN or blood total leukocyte counts or in the number of CD4^+^ and CD8^+^ T cells or B cells in steady state (Supplemental Figure 2, A and B). Lymphatic tissue, such as mesenteric LNs (mLNs) and Peyer’s patches (PPs), which normally has higher activity in the steady state, showed no differences in T cell memory, effector, or naive cell populations or in total or marginal zone B cells (Supplemental Figure 2, C and D).

### Impaired skin inflammation in the absence of MC-derived RANKL.

We studied the role of MC-derived RANKL in skin inflammation using the contact hypersensitivity (CHS) mouse model, which is a T cell–mediated, delayed-type hypersensitivity response. Sensitization with the hapten 1-fluoro-2,4-dinitrobenzene (DNFB) on back skin results in hapten-specific T cell expansion in inguinal LNs, which then infiltrate the ear skin upon DNFB challenge and initiate skin inflammation. Importantly, we found that both the ear swelling and infiltration of leukocytes, particularly of CD8^+^ T cells and neutrophils, was massively impaired in MCΔRANKL as compared with MCWT mice 24 hours after challenge (Figure 1, A and B). At the same time, the numbers of all blood lymphocyte populations, CD4^+^ and CD8^+^ T cells, and B cells were significantly reduced by half in MC^ΔRANKL^ mice as compared with MC^WT^ control mice, whereas neutrophil numbers remained unchanged (Figure 1, C and D). Upon first hapten encounter on back skin, skin DCs process haptenized self-proteins and migrate to inguinal dLNs to prime hapten-specific T cells (12). Given our previous observations of MC-DC communication (14, 19), we questioned whether the reduced skin and blood lymphocyte numbers may have resulted from impaired DC migration. Using an FITC-induced CHS assay, which allowed tracking of FITC^+^ DCs (18), we found that both total DC numbers and FITC^+^ DCs numbers in inguinal LNs were not altered in MC^ΔRANKL^ mice compared with MC^WT^ mice (Figure 1E). Moreover, epidermal thickness during sensitization was not altered on day 1 or day 4 after sensitization with DNFB, and transepidermal water loss was also not different between MC^ΔRANKL^ and MC^WT^ mice (Supplemental Figure 3, A and B).

### Lack of MC-derived RANKL leads to inguinal LN hyperplasia upon CHS elicitation.

Surprisingly, we found that inguinal LNs draining the sensitized back skin showed dramatic hyperplasia 24 hours after challenge (Figure 1F). The total numbers of CD3^+^ T cells, CD4^+^ T cells, CD8^+^ T cells, and B cells were massively increased in MC^ΔRANKL^ mice compared with MC^WT^ mice (Figure 1G). By normalizing the number of lymphocytes in MC^ΔRANKL^ mice to that in MC^WT^ mice, we found that all lymphocyte populations had at least doubled (Figure 1H).

Given the importance of RANKL for LN anlagen, development, and growth, we aimed to evaluate any structural dysregulation or maladaptation of inguinal LNs in the context of this LN hyperplasia. Therefore, we performed whole-mount staining of all intact inguinal LNs, modified according to Li et al. (20), followed by tissue clearing and 2-photon microscopy of all cleared LNs (Supplemental Figure 4A). First, LN 3D projection and optimal sectioning in the midpoint of 3D images proved respective whole-mount staining efficiency and specificity of T and B cell areas, blood vessels, and lymph vessels (Supplemental Figure 4, B and C). In order to not only visualize the respective zones but also quantify structural parameters, whole LNs were scanned, and LN volume as well as T cell and B cell areas were reconstructed using the Imaris surface tool with the machine learning function, based on fluorescence intensity, and quantified as volumes (Figure 2, A–D). The sizes of the whole inguinal LNs, as well as the T cell and B cell zones separately, were found to be consistently increased, confirming significant LN hyperplasia and an increase in all lymphocyte subsets in the absence of MC-derived RANKL (Figure 2, B–D). However, the ratios of T cell to B cell zone volume, as well as of T cell and B cell zones to the total inguinal LN volume, were not significantly different, showing that the inguinal LNs were generally enlarged without a specific effect on some compartments (Figure 2E). Using the filament tracer for blood vessel reconstruction and quantification, we found that the vessel volume and diameter were not increased in MC^ΔRANKL^, while the total segment length was increased, showing more outstretched vessels correlating to the increased LN size (Figure 2F). Overall, no visible structural defects or maladaptations were observed except for the LN enlargement.

Given how important fibroblastic reticular cells (FRCs) are for the development and expansion of the LN, we investigated whether steady-state alterations existed upon MC-RANKL deletion (21). We tested inguinal LNs for FRC-related chemokine expression by quantitative PCR (qPCR). We found that the main FRC homeostatic chemokines *Ccl19* and *Ccl21*, as well as *Cxcl9*, *Cxcl10*, and *Cxcl13*, were unaltered in MC^ΔRANKL^ mice (Supplemental Figure 5A). To address whether LN enlargement reflected a broader preexisting structural maladaptation, we performed additional multi-epitope ligand cartography (MELC) analysis of LN organization. This included the spatial localization of major lymphocyte populations in relation to the FRC network, with quantitative assessment of the distances of CD4^+^ T cells, CD8^+^ T cells, and B cells from FRCs, as well as their relative cellular frequencies within the LN. Importantly, we found that none of these parameters differed between groups. Thus, despite the clear increase in LN size, the relative distribution of major lymphocyte populations, their positioning with respect to the FRC scaffold, and their overall representation in the tissue remained preserved. Together with the unchanged expression of key lymphoid chemokines, these data argue against a preexisting defect in LN stromal organization or lymphocyte compartmentalization as the cause of the observed LN hyperplasia. (Supplemental Figure 5, B and C).

To determine whether the LN hyperplasia was driven by a lymphocyte-intrinsic inability to expand or migrate, we isolated lymphocytes from the spleen and LNs of sensitized WT mice at day 6 after DNFB, CFSE-labeled them, and adoptively transferred them into sensitized MC^ΔRANKL^ and MC^WT^ mice 1 day after DNFB sensitization of recipient mice. Five days later, we analyzed the inguinal LNs of the recipient mice (Figure 3A). As expected, endogenous (CFSE^–^) lymphocyte numbers were markedly increased in MC^ΔRANKL^ mice compared with MC^WT^ mice, excluding any transfer-induced effects on inguinal LN hyperplasia (Figure 3B). Of note, we observed a massively increased number of transferred CFSE^+^ WT lymphocytes in inguinal LNs of MC^ΔRANKL^ recipient mice compared with MC^WT^ mice, again affecting all lymphocyte populations, including CD4^+^ and CD8^+^ T cells and B cells, indicating that the transferred WT lymphocytes reached the inguinal LNs and underwent expansion but were retained in the absence of MC-derived RANKL (Figure 3C). Collectively, these data demonstrate that the LN hyperplasia was not driven by lymphocyte -intrinsic effects but rather was dictated by the tissue, without any developmental or structural LN defects.

Transient LN hyperplasia and blood lymphopenia persistently impaired skin inflammation. Next, the kinetics of blood lymphopenia and inguinal LN hyperplasia were determined at days 4, 6, and 10 after sensitization and were found to be transient (Figure 3, D and E). More specifically, inguinal LNs showed increased cellularity on day 4, with no differences yet observed between MC^ΔRANKL^ and MC^WT^ mice. On day 6, the highest lymphocyte count was reached, with the hyperplasia in MC^ΔRANKL^ mice showing nearly 2-fold more lymphocytes than in MC^WT^ mice. On day 10, inguinal LN cellularity was reduced to the same level in both MC^WT^ mice and MC^ΔRANKL^ mice compared with day 4, indicating lymphocyte egress (Figure 3D). We observed transient effects in the blood as well, since lymphocyte numbers were similar between MC^ΔRANKL^ and MC^WT^ mice at day 4 but reduced at day 6 (Figure 3E). Moreover, the blood lymphopenia was reversed by day 10, with significantly more CD4^+^ and CD8^+^ T cells and B cells in the blood of MC^ΔRANKL^ mice compared with that of MC^WT^ mice (Figure 3F), indicating a delayed onset of lymphocyte egress in the absence of MC-derived RANKL. Given the delayed lymphocyte egress in MC^ΔRANKL^ mice, we also examined the kinetics of skin inflammation and ear skin lymphocyte infiltration after challenge (Figure 4, A and B). In MC^WT^ mice, the ear swelling peaked at approximately 40% at 48 hours after challenge with a rapid decline thereafter. In MC^ΔRANKL^ mice, ear swelling was significantly reduced in the first 48 hours compared with MC^WT^ mice, with a peak of only approximately 20% at day 3, and declined thereafter similarly to the ear swelling seen in MC^WT^ mice (Figure 4A). Surprisingly, the reduced lymphocyte infiltration at 24 hours (Figure 1A) was reversed at 48 hours, thus showing significantly higher numbers of CD4^+^ and CD8^+^ T cells in ear skin (Figure 4B). This finding is in line with the reversed lymphocyte levels in the blood at later time points but contrasted with the impaired ear swelling. We therefore specifically determined the extravasation of lymphocytes to exclude artifacts of blood microvessel lymphocytes instead of those in skin tissue. Blood circulating cells were first stained in vivo by i.v injection of phycoerythrin-conjugated (PE-conjugated) anti-CD45 mAb, and following ear skin digestion, all leukocytes were counterstained with PercP-Cy5.5–conjugated anti-CD45 mAb (Figure 4C). Hence, skin lymphocyte numbers were increased in MC^ΔRANKL^ mice compared with MC^WT^ mice (Figure 4D), and the extravasation capacity determined as the ratio of circulating and tissue lymphocytes was not altered (Figure 4E). In contrast to increased skin lymphocyte numbers, the ear skin concentration of the cytokines IFN-γ, TNF, IL-4, and IL-2 were not altered in MC^ΔRANKL^ mice compared with MC^WT^ (Figure 4F).

Peripheral skin MC production of RANKL is crucial for lymphocyte egress from distant LNs. Given the LN hyperplasia observed in the absence of MC RANKL and the strong expression of RANKL by MCs in the steady state (Figure 5A), we sought to determine whether MCs in skin or LNs are the key source. MCs were depleted only locally in the ear skin of Mcpt5-Cre iDTR mice by intradermal (i.d.) diphtheria toxin (DT) injection (18), and reconstituted with MCs from either MC^ΔRANKL^ or MC^WT^ mice. Efficient MC depletion and reconstitution was confirmed by microscopy (data not shown), and CHS was performed 2 weeks after reconstitution. Consequently, only MCs in the DNFB-challenged ear skin lacked RANKL production, while all other MCs, in sensitized back skin and LNs as well, are RANKL-proficient (Figure 5B). Intriguingly, absence of RANKL in ear skin MCs only resulted in reduced ear swelling upon DNFB challenge (Figure 5C), accompanied by impaired infiltration of CD4^+^ and CD8^+^ T cells and neutrophils (Figure 5D). Moreover, mice lacking RANKL in only ear skin MCs still exhibited the massive inguinal LN hyperplasia with increased numbers of all CD4^+^ and CD8^+^ T cells and B cells 24 hours after challenge, as well as simultaneous blood lymphopenia with a significant reduction in CD4^+^ and CD8^+^ T cell numbers compared with mice reconstituted with WT peritoneal cell–derived MCs (PCMCs), as observed in MC^ΔRANKL^ mice (Figure 5, E and F). These data indicate that RANKL release by ear skin MCs upon CHS challenge drives a long-range effect toward the distant back skin inguinal dLNs that is a prerequisite for timely lymphocyte egress.

MC-derived RANKL triggers lymphocyte egress by inducing an early blood sphingosine-1–phosphate (S1P) burst. Next, we aimed at deciphering the mechanism underlying the remote RANKL effect from ear skin to distant inguinal LNs. RANKL has been reported to stimulate the secretion of S1P (22), a signaling molecule initiating lymphocyte migration (23, 24). Since lymphatic endothelial cells (LECs) are known to be an important source of S1P, we next investigated whether RANKL can stimulate S1P release by LECs. To address this, cultured ear skin LECs were treated either with recombinant RANKL or with supernatants from activated PCMCs. PCMC supernatants were collected after either early activation for 2 hours or prolonged activation for 24 hours. Both recombinant RANKL and supernatants from activated MC^WT^ PCMCs induced S1P release by LECs. In contrast, supernatants from activated MC^ΔRANKL^ PCMCs failed to induce this response. These findings suggest that MC-RANKL is sufficient and required in the MC supernatant to promote S1P release by LECs. (Figure 6A). Moreover, although similar in the steady state, serum S1P steadily increased up to day 4 after sensitization in MC^WT^ mice and to a significantly lesser degree in MC^ΔRANKL^ mice, but serum S1P had dropped to basal levels by day 6 for both genotypes. (Figure 6B). This indicates that MC RANKL was involved in the regulation of the S1P response during CHS. By analyzing the kinetics of serum S1P levels upon CHS challenge, we found early onset of S1P release only 2 hours after DNFB challenge with a peak at 24 hours and a decline thereafter until 48 hours. Importantly, the early S1P production 2 hours after DNFB compared with vehicle-treated mice was completely missing in MC^ΔRANKL^ mice, with levels even below those of the vehicle-treated control group (unspecific skin irritation), whereas the delayed onset was more quickly reduced to significantly lower levels at 48 hours (Figure 6B). Surprisingly, i.d. injection of RANKL alone was not sufficient to induce the S1P burst at 2 hours, indicating a preconditioning during sensitization, a synergistic effect of MC RANKL with other mediators, or a stabilization of RANKL within MC secretory granules and a focused degranulation (Figure 6C). Nevertheless, since the early S1P burst 2 hours after DNFB in MC^WT^ mice may have been triggered by the immediate release of preformed MC-derived RANKL, we speculated that the phenotype of inguinal LN hyperplasia and blood lymphopenia in MC^ΔRANKL^ mice could be rescued by i.v. injection of recombinant S1P 2 hours after DNFB challenge (Figure 6D). Hence, CHS was performed in MC^WT^ and MC^ΔRANKL^ mice, and MC^ΔRANKL^ mice received an i.v. injection of 500 ng recombinant S1P (simulating the S1P concentration in MC^WT^ mice) or PBS as a control 2 hours after DNFB and were compared with MC^WT^ mice. Intriguingly, the lymphocyte infiltration into the ear skin was significantly increased compared with PBS control mice, reaching the levels seen in MC^WT^ mice (Figure 6E). In line with this observation, the single S1P injection was sufficient to significantly reverse both the inguinal LN hyperplasia and blood lymphopenia of CD4^+^ and CD8^+^ T cells and B cells 24 hours after challenge to levels in MC^WT^ mice (Figure 6, F and G). These results reveal that RANKL released peripherally by skin MCs was inducing lymphocyte egress in distant LNs upon skin inflammation through a remote mechanism involving early S1P release into the bloodstream.

## Discussion

There is increasing evidence that peripheral MCs are involved in LN hyperplasia in inflammatory conditions (12). For example, TNF released from skin MCs upon bacterial challenge is rapidly detectable in dLNs (25, 26). Moreover, MCs play a pivotal role in TNF-independent LN expansion upon i.d. bacterial peptidoglycan injection (26, 27). Kunder et al. showed that after peripheral MC degranulation, released MC granules can enter the lymphatic vessels and reach the dLNs, thereby eliciting profound LN hypertrophy, whereas no MC activation in the LNs was observed (28). In addition, we were able to show that, upon LPS- or hapten-induced skin inflammation, released, intact MC granules were taken up by skin-resident DCs, which promotes DC maturation and migration to dLNs, thereby priming T cells (29). Hence, mechanistically, MC-DC communication in the skin, involving both MC granule effects and physical intercellular interaction (13), as well MC mediator drainage from the skin through lymphatics, both contribute to remote LN hyperplasia. However, MCs release a plethora of proinflammatory mediators which, in addition to TNF, may have an effect on LN conditioning in one way or another. We and others have proven that MCs are a relevant source of RANKL (30, 31). However, although RANKL overproduction in hair follicles has been found to cause significant LN hyperplasia (32), the role of MC-derived RANKL in adaptive skin inflammation remains to be investigated.

In this study, we show for the first time to our knowledge, that skin MC–derived RANKL was involved in the conditioning of distant LNs upon skin inflammation. While the local inflammatory response was impaired upon hapten challenge in the absence of MC-derived RANKL, inguinal LNs showed massive hyperplasia, involving all the lymphocyte populations that expanded upon hapten sensitization. Notably, this effect was observed after hapten challenge on ear skin, but in the distant inguinal dLNs that drain lymph from the sensitized back skin. Importantly, we were able to prove that peripheral skin MCs, but not MCs in the LNs, were responsible for this long-distance communication phenomenon by a local ear skin MC depletion and reconstitution with either WT or RANKL-deficient MCs. A long-distance effect was further supported by the finding that DC migration to inguinal LNs upon back skin sensitization was not affected. The massive inguinal LN hyperplasia was accompanied by simultaneous blood lymphopenia, indicating that lymphocytes expanded as expected but failed to egress from inguinal LNs to the blood. This could have been caused by direct MC RANKL effects on the LNs or by indirect effects via cellular mediators.

Skin MCs are well known to initiate recruitment of innate, but also adaptive, immune cells such as T cells (8). For example, direct MC degranulation into the bloodstream is crucial to prime and recruit neutrophils in CHS (15). Hence, MC granules may reach distant organs via the bloodstream and therefore exert long-range effects. The plethora of MC mediators, in conjunction with their ability to promote adaptive immune cell expansion by their effects on DC migration and maturation, make it difficult to discriminate between their effects on T cell priming versus recruitment. To exclude MC effects on T cell priming or T cell–intrinsic inabilities to egress from LNs, we adoptively transferred WT T cells into mice lacking MC RANKL and found that these cells reached the inguinal LNs and expanded properly, but were unable to leave the inguinal LNs as well. This finding supported the hypothesis of a missing egress signal, originating from peripheral MCs, that is independent of the T cell–priming and expansion capacity.

When we studied the kinetics of the inflammatory response, we noticed lymphocyte egress from distant inguinal LNs at later time points coinciding with a reversal of the blood lymphopenia. The lack of MC RANKL–controlled lymphocyte egress could either be compensated by other cells or overcome due to organ-based limits of cell hyperplasia. Importantly, although lymphocytes eventually reached the challenged ear skin at later time points, their delayed arrival was associated with a weaker inflammatory response. This suggests that the early phase of cell recruitment is critical for setting the inflammatory tone of the tissue. Even though total leukocyte numbers were increased 48 hours after DNFB challenge, the reduced infiltration at 24 hours may have already limited the local production and amplification of inflammatory mediators. In particular, delayed T cell entry could result in reduced availability of cytokines and chemokines that are needed to sustain inflammation and support the recruitment of additional effector cells. At the same time, MC-derived proteases may further shape this environment by processing or degrading cytokines and chemokines after degranulation. Since MC-specific RANKL deficiency did not affect MC degranulation, the phenotype is unlikely to reflect a general defect in granule release. Rather, our data suggest that MC-derived RANKL contributed to the timely recruitment of lymphocytes to inflamed skin and thereby influenced the subsequent inflammatory cascade. In line with this, neutrophil infiltration into the challenged skin was also delayed, despite normal mobilization of neutrophils to the blood, indicating that the defect lay mainly at the level of tissue recruitment rather than systemic availability. Consequently, our data showed that early RANKL signaling induced by peripheral skin MCs was critical for timely lymphocyte egress from distant inguinal LNs, which, in turn, was crucial for the proper onset of inflammation.

When investigating the long-range signaling mechanism, we found that an early rise in serum S1P levels just 2 hours after DNFB challenge was abolished in the absence of MC-derived RANKL. Importantly, i.v. injection of the same S1P amount at this time point restored both the inguinal LN hyperplasia and blood lymphopenia, as well as lymphocyte infiltration into the skin to normal levels. It has been reported previously that S1P is crucial for controlling lymphocyte egress from lymphoid tissues. The S1P concentration is always higher in the blood and low in peripheral tissues. The S1P receptor 1 (S1P_1_) is internalized in circulating lymphocytes, given the high S1P concentration and reexpressed in S1P-poor tissues, allowing for a gradient-promoting egress from secondary lymphoid organs to the blood (23). Adoptively transferred lymphocytes lacking the S1P_1_ can enter but not leave secondary lymphoid organs (33, 34). Deletion of the S1P lyase increases total S1P concentrations in the LN and blocks lymphocyte egress. Similarly, deletion of both S1P kinases in LECs blocks lymphocyte egress (35, 36). Therefore, the S1P spike we observed in the blood after DNFB challenge in MC^WT^, but not MC^ΔRANKL^, mice, seemed to be acting as a lymphocyte egress signal, whereas the early time point indicates that preformed RANKL contained in the MC granules may be responsible.

Unexpectedly, intradermal injection of recombinant RANKL alone did not reproduce the rapid S1P burst observed 2 hours after challenge. Rather, this indicates that RANKL is unlikely to act as an isolated trigger in vivo. Instead, the response may require prior tissue conditioning during sensitization or a synergistic effect with other MC-derived mediators. The marked dysregulation of serum S1P in the absence of MC RANKL during sensitization, despite its equilibration to normal levels just before challenge, may play an important role in priming the subsequent S1P spike after challenge. Moreover, the local delivery of RANKL during focused degranulation can also be important. In this context, storage within MC secretory granules may stabilize RANKL (37) and enable its targeted and concentrated release into perivascular or lymphatic niches. Importantly, MC RANKL could also act through direct MC interaction and communication with S1P controlling cells in the above niches. Thus, MC-derived RANKL appears to regulate early S1P release in a context-dependent manner rather than through simple soluble exposure alone. Importantly, we were able to show that in vitro–cultured skin LECs responded to recombinant and MC RANKL with the production of S1P. MC RANKL may have been acting on skin LECs or by directly reaching the LN through the circulation. Thus, it is plausible that a MC-LEC communication axis is involved in controlling lymphocyte egress from distant LNs during skin inflammation.

Nevertheless, the contribution of additional cells cannot be excluded. Although inflammatory monocytes have been shown to raise LN S1P levels, thereby retaining lymphocytes in the tissue (38), and are recruited by MCs (12), the 2-hour time point is too early for both a substantial monocyte infiltration and their S1P synthesis. Therefore, we speculate that the S1P spike results from MC RANKL effects on blood vessel endothelial cells (BECs). BECs express the receptor RANK, and RANKL stimulation promotes the survival and proliferation of BECs, thus inducing angiogenesis (39, 40). Moreover, BECs are one of the main controllers of serum S1P levels (41, 42). Given the ability of perivascular MCs to pass the vessel wall and degranulate directionally into the bloodstream (15), RANKL contained in MC granules may activate BECs systemically, even in distant blood vessels to immediately raise blood S1P concentrations upon inflammatory insult. Moreover, S1P may be released by the MCs themselves, which are a known source of S1P, in an autocrine manner (43).

This study demonstrates that skin MC–derived RANKL is essential for lymphocyte egress from distant LNs via a mechanism involving S1P signaling. In the absence of MC-derived RANKL, expanded lymphocytes do not egress from LNs in a timely manner upon skin inflammatory insult, resulting in massive LN hyperplasia and severe blood lymphopenia. Moreover, delayed lymphocyte egress and skin infiltration lead to attenuated CHS symptoms. Our results highlight the ability of peripheral MCs to act remotely and the importance of skin MC–derived RANKL in regulating lymphocyte egress and the initiation and progression of inflammation, with substantial implications for the treatment of inflammatory diseases.

## Methods

### Sex as a biological variable.

Our study examined male and female mice, and similar findings are reported for both sexes.

Additional details on Methods are provided in the Supplemental Methods. For additional information on reagents, software, mouse lines, and chemicals please refer to Supplemental Table 1.

### Mice.

Mcpt5-Cre iDTR [Tg(Cma1-Cre)ARoer x Gt(ROSA)26Sor^tm1(HBEGF)Awai^] and Mcpt5-Cre RANKL^FL/FL^ Tg(Cma1-Cre)ARoer x B6.129-Tnfsf11^tm1.1Caob^/J mice were housed at the Central Animal Facility, Medical Faculty, Otto von Guericke University Magdeburg, under specific pathogen–free conditions. All lines were on a C57BL/6 background. In all experiments, mice were used at the age of 8–12 weeks. Littermates were used as controls. For the Mcpt5-Cre line, Cre recombinase expression has been previously proven to be specific for connective tissue type MCs using a Cre-induced EYFP reporter line, whereas no expression was found in mucosal MCs or in other immune cells with the exception of a small fraction of NK cells (18).

### Proof of RANKL KO.

The deletion on the *Tnsf11* happens in exons 3, 4, and 5 (44). Therefore, exons 1 and 2 are still expressed. Exon 1 encodes the intracellular and transmembrane part of the RANKL protein, and exon 2 encodes the first part of the extracellular domain of the protein. Without exons 3, 4, and 5, which code for the remaining extracellular domain, the RANKL protein cannot signal through the RANK receptor. However, proof of the KO needs to be performed with an antibody that binds the domain encoded by exons 3, 4, and 5. Polyclonal antibodies tend to detect the encoded regions of exons 1 and 2 and therefore also give signal in the MC^ΔRANKL^. The antibody used in this study was the CD254 antibody from BioLegend (clone IK22/5, RRID AB_2303587).

### Contact hypersensitivity.

Mice were sensitized with 100 μL 0.5% DNFB (w/v) in acetone/olive oil (4:1) on the shaved back and challenged 6 days later with 20 μL 0.2% DNFB (10 μL on each side of the ear). Vehicle control mice were treated only with the solvent during the sensitization and challenge steps. Ear thickness was measured before and at several time points after challenge with an engineer’s micrometer (Mitutoyo). Ear swelling as an indicator of skin inflammation was calculated as the percentage increase compared with prechallenge ear thickness.

For FITC-induced CHS, mice were sensitized by epicutaneous application of 100 μL FITC (0.5% w/v in dibutyl-phtalate/acetone 1:1) on the shaved back. Forty-eight hours after sensitization, inguinal LNs were isolated, and DCs were analyzed by flow cytometry. Transepidermal water loss was measured on the sensitized back skin with a Tewameter TM300 (Courage and Khazaka), which sampled 30 consecutive measurements of each skin location, the mean of which was recorded. Moreover, 3 different spots were measured from the back of each mouse, and the mean was used for every time point. The measurements were taken in a room with controlled temperature and humidity. More specifically, ambient humidity ranged between 43% and 52% and the room temperature (RT) between 21°C and 23^o^C.

### Adoptive transfer.

CFSE-labeled splenocytes were adoptively transferred in 37°C prewarmed, sterile PBS by intravascular injection through the retro-orbital sinus.

### Cell preparation and flow cytometry.

For preparation of skin cell suspensions, samples of ear skin were cut into small pieces and digested in RPMI 1640 (PAN Biotech) containing 0.025 mg/mL Liberase (Roche), 396 U/mL DNase I, and 0.5 mg/mL hyaluronidase at 37°C, 1,400 rpm for 1 hour. Samples were passed through a 40 mm sieve, and the cell suspension was washed twice with PBS.

For flow cytometric analysis, isolated cells were incubated with an anti-FcγRIII/II antibody (clone 93) to block unspecific staining. Thereafter, cells were resuspended in PBS with 2% BSA, and 1 × 10^6^ to 2 × 10^6^ cells were stained with fluorochrome-conjugated mAbs in 100 μL staining solution for 15 minutes at 4°C. Cell suspensions were washed twice and resuspended in 200 μL PBS and 2% BSA. Matched fluorescence minus one controls (FMOs) were used to assess the level of background fluorescence in the respective detection channel. Flow cytometric analysis was performed on a BD LSR Fortessa and analyzed with FlowJo software.

### Preparation of single-cell suspensions from the spleen, mLNs, and PPs and analysis via flow cytometry.

To prepare single-cell suspensions, mLNs were first cleared of adipose tissue, and mLNs and PPs were finely minced with a scalpel. Next, spleen, mLNs, and PPs were forced through 100 μm filters, and cells were centrifuged (400*g*, 5 minutes, RT). Splenocytes were subjected to ammonium-chloride-potassium lysis to remove erythrocytes, followed by quenching with RPMI 1640 (PAN Biotech) plus 10% (v/v) FCS (MilliporeSigma). After centrifugation, final cell suspensions were filtered through a 40 μm filter. The following antibodies were used for staining: anti–mouse CD3 (17A2), CD8a (53–6.7), CD11c (N418), CD19 (6D5), CD45 (30-F11), CD62L (MEL-14), F4/80 (BM8), anti–mouse CD4 (GK1.5), CD11b (M1/70), CD44 (IM7), Ly6G (1A8), CD21/35 (eBio8D9), CD23 (B3B4), Foxp3 (FJK-16s), IgD (11-26c), and IgM (II/41). Cells were first stained for viability using Fixable Viability Dye eF780 for 20 minutes at 4°C. Next, Fc receptors were blocked with anti–mouse CD16/32 (purified from 2.4G2 ATCC HB-197) for 10 minutes at 4°C prior to staining with surface antibodies for 30 minutes at 4°C. For intranuclear staining, samples were fixed and permeabilized using the Foxp3/Transcription Factor Staining Buffer Set (Miltenyi) for 30 minutes or overnight at 4°C, followed by a 60-minute incubation with intranuclear antibodies at 4°C. Samples were measured on a BD LSRFortessa and analyzed with FlowJo software (version 10). B cell subsets were gated according to Figure 2 in ref. 45.

### Reverse transcriptase PCR and real-time qPCR.

One inguinal LN was homogenized in 200 μL TRIzol (Thermo Fisher Scientific) using CK14 tubes and the Precellys 24 homogenizer (Bertin Technologies) according to the manufacturer’s protocol. Cultured PCMCs were resuspended in 1,000 μL TRIzol. Following chloroform addition, total RNA was isolated according to the manufacturer’s protocol and quantified via NanoDrop (Thermo Fisher Scientific) spectrophotometry. RNA was subsequently reverse transcribed using random hexamer primers and the Advantage RT-for-PCR kit according to the manufacturer’s instructions (Takara Bio). Real-time qPCR (RT-qPCR) was performed using TaqMan Gene Expression Master Mix and TaqMan Gene Expression Assays (Thermo Fisher Scientific): Ccl19 (FAM-MGB probe Mm00839967 g1), Ccl21 (FAM-MGB probe Mm03646971 gH), Cxcl9 (FAMMGB probe Mm000434946 m1), Cxcl10 (FAM-MGB probe Mm00445235 m1), Cxcl13 (FAM-MGB probe Mm00444534 m1), and Hprt (FAM-MGB probe Mm00446968 m1). All quantitative analyses were performed in triplicate on the qTOWER³ cycler (Analytik Jena), and relative expression levels were determined using the ΔCT method.

### Whole-mount LN staining and microscopy.

Mice were anesthetized with isoflurane and injected i.v. with anti–CD31 AF594 mAb for the blood vessel stain. Five minutes later, mice were sacrificed and inguinal LNs were collected. The LNs were fixed overnight at 4°C in 1% PFA fixation buffer (0,7% w/v Na_2_ hours PO_4_, 1,5% w/v lysine, 0,2% w/v NaIO_4,_ 1% PFA in ddH_2_O). Afterwards, the LNs were incubated for 5 days in blocking buffer (0.02% goat serum, 1% BSA, 0.3% Triton X-100, 0.5 μg/mL streptavidin in PBS), followed by a 7-day primary antibody staining in blocking buffer, all at RT. T cell zones were stained with a rat monoclonal anti–mouse CD8 (53-6.7) Alexa Fluor 488 conjugate, B cell zones with a rat monoclonal anti–mouse B220 (MAR-1) Alexa Fluor 647 conjugate, and lymphatic structures with a rat monoclonal anti–mouse Lyve1 (ALY7) Brilliant Violet 421 conjugate. The antibody staining step was followed by a 1-day staining step with streptavidin BV421. Next, LNs were washed for 1 day in wash buffer (0.5% thioglycerol, 0.2% Triton X-100 in PBS) at RT, followed by dehydration with 50% ethanol for 2 hours, 70% ethanol overnight, and absolute ethanol for 2 hours. Subsequently the LNs were cleared in 100% ethyl cinnamate by shaking at RT for 15 minutes and stored in 100% ethyl cinnamate until microscopy. Whole LNs were analyzed by 2-photon laser-scanning microscopy and fully submerged in ethyl cinnamate as described below.

Two-photon laser scanning microscopy was performed with an upright, fixed-stage Leica Stellaris 8 microscope with simultaneous detection via 4 external spectral non-descanned detectors. Illumination was performed at 840 nm and 980 nm with a MaiTai Deep See and an Insight X3 TiSa laser (both from MKS Spectra-Physics). A Leica HC FLUOTAR L 16×/0, 60 IMM CORR VISIR objective was used to image the LNs, which were immersed in an ethyl cinnamate filled glass Petri dish. Bidirectional tile scans (25% overlap) of the LN were recorded as *Z*-stacks (Dz = 5 mm) encompassing the entire LN with an *x*-*y* resolution of 1,024 × 1,024 pixels per single image at 600 Hz scan speed with a line average of 4. Data were visualized as surface-rendered *Z*-stacks or single *Z*-planes. Raw data were reconstructed, analyzed, and quantified using Imaris. For the blood vessel analysis, the Imaris filament tracer was used, while the T cell zones, B cell zones, and lymphatic tissues were reconstructed with the surface tool. The volume of different LN compartments and blood vessel parameters, such as diameter and segment length, were calculated according to the surfaces and filaments.

### Whole-mount back skin staining and microscopy.

For whole-mount staining and light-sheet imaging of back skin samples, mice were sacrificed, and a square of shaved skin was cut from the back. The skin samples were treated with hair removal cream for 1 minute and washed in PBS. Samples were washed in PBS and permeabilized in permeabilization buffer (20% DMSO, 0.3 M glycine, 0.2% Triton X-100, 2.5 μg/mL streptavidin in PBS) overnight at 4°C, followed by decolorization in 25% Quadrol (w/v) in distilled H_2_O overnight at RT. After this, the samples were further permeabilized in NaOH solution (0.1 M NaOH plus 0.01% Triton-X in PBS) for 20 minutes at 4°C. Samples were decolorized as described above, dehydrated by an increasing ethanol series, and bleached in bleaching solution (methanol/DMSO/30% H_2_O_2_ in a 1:4:4 ratio) overnight at 4°C. The samples were rehydrated, and whole-mount staining was performed as described above. Primary antibody staining was performed with a rat anti–mouse CD31 (MEC13.3) mAb and an avidin Alexa Fluor 488 conjugate for 7 days. Secondary staining was performed with an anti–rat DyLight 800 secondary antibody and Sytox DeepRed nucleus dye for 6 days. Alexa Fluor 633 hydrazide was added to the staining solution for 24 hours. Finally, the samples were embedded and cleared as described above and imaged with a Miltenyi Biotec UltraMicroscope Blaze light-sheet microscope.

### Dermal sheet staining and microscopy.

To analyze MC numbers and localization in the ear skin, ears were isolated and split into dermal sheets. Samples were fixed overnight in 1% PFA in PBS at 4°C with mild shaking. Afterwards, samples were permeabilized on ice in a methanol/aceton solution (1:1) for 10 minutes per side, washed for 10 minutes in PBS, and incubated in 100 mM glycine for 10 minutes for fluorescence signal enrichment. After washing, blocking was performed with 0.5 μg/mL streptavidin in PBS for 60 minutes at RT. MCs were stained with an avidin-TexasRed (Molecular Probes) conjugate for 1 hour. For primary antibody staining, samples were washed 4 times in PBS and blocked in blocking buffer (2% serum matching the secondary antibody, 50 mM glycine, 0.05% Tween 20, 0.1% Triton X-100, 1% BSA) for 60 minutes. Subsequently, samples were stained with a rat anti–mouse monoclonal (eBioV.7C7) endomucin–eFluor 660 conjugate, a mouse anti–mouse monoclonal (1A4) αSMA–eFluor 660 conjugate, and a RANKL polyclonal antibody overnight at 4°C, followed by 4 washings in 0.1% Tween 20 in PBS. Secondary staining was performed with a goat anti–rat Alexa Fluor 647 conjugate and a goat anti–rabbit Alexa Fluor 488 conjugate in 0.1% Tween 20 in PBS for 1 hour at RT. After 3 washings, samples were stained with DAPI for 15 minutes at RT, washed 3 times, and air-dried. Finally, the samples were mounted onto an object slide for microscopy in Vectashield Antifade Mounting Medium (Biozol), and the coverslip was sealed with nail polish. Imaging was performed within 1–4 days using an inverted Leica TCS SP8 confocal laser-scanning microscope. For imaging of whole dermal sheets, illumination was performed via a Leica HC PL APO ×20/0.75 IMM CORR CS2 oil dipping lens. The dermal sheets were imaged in tile scans and stitched afterwards with the Imaris stitcher (version 10.1.1). Images were recorded as 12 bit *Z*-stacks with a resolution of 512 × 512 pixels, a depth of 200–250 μm, and a *Z* spacing of 5 μm. For imaging of RANKL^+^ MCs, samples were illuminated via the ×40 oil objective. Images were recorded with a *Z* spacing of 0.5 μm.

### Quantification of extravasation.

To analyze lymphocyte extravasation from the blood vessels into the ear skin tissue (dermis), mice were challenged with 2% DNFB for 48 hours. Subsequently, CD45 PE antibody was injected i.v. after mice were anesthetized with an i.p. injection of ketamine 0.1 mg/kg, and (Elanco) 10 mg/kg. In this way, all lymphocytes located in the blood vessels were labeled with PE. Five minutes after the injection of CD45 PE, the mice were sacrificed, ears were collected and digested, and a single-cell suspension was prepared and stained for FACS. Lymphocytes that were located inside the blood vessels were therefore stained with the CD45 PE antibody, and lymphocytes in the skin tissues were able to be separated by a second staining with CD45 PercP-Cy5.5 that was performed ex vivo.

### Culturing of PCMCs.

Peritoneal MCs were harvested by peritoneal lavage with 5 mL ice-cold PBS and cultured in RPMI complete medium containing 10% FCS, 1% penicillin/streptomycin, 50 μM β-mercaptoethanol, and 1% sodium pyruvate. The medium was supplemented with 10 ng/mL IL-3 and 30 ng/mL stem cell factor (SCF). The medium was changed on days 2, 5, and 8, and the cells were used for reconstitution on day 10. From day 5, the SCF concentration was switched to 10 ng/mL.

### PCMC degranulation assay.

For quantification of MC degranulation, 1 × 10^5^ PCMCs were seeded in RPMI complete medium. For DNP/IgE stimulation, PCMCs were seeded in 150 ng/mL anti–DNP IgE for sensitization. On the next day, sensitized PCMCs were stimulated with 100 ng/mL DNP-BSA and untreated PCMCs with 500 ng/mL calcium ionophore A23187, 500 μM ATP, or 50 ng/mL IL-33 in a total volume of 125 μL RPMI complete medium for 10 minutes, respectively. Then, the cell culture plate was placed on ice for 5 minutes and centrifuged (365*g*, 5 minutes, 4°C) before the cells were stained for flow cytometry as described above.

### MC depletion and local reconstitution of ear skin.

In Mcpt5-Cre inducible diphtheria toxin receptor mice, MCs were locally depleted in the ear skin by a single injection of 100 ng DT in 20 μL sterile PBS. Five days after MC depletion, the ears were reconstituted with 1 million PCMCs in 20 μL sterile PBS per ear. The cells were allowed to repopulate the tissue for 2 weeks before the induction of CHS.

### Automated multidimensional fluorescence microscopy by MELC.

MELC was performed as described previously (46). Briefly, peripheral LNs from RANKL WT and KO mice were embedded into in Tissue-Tek O.C.T. Compound (Sakura Finetek), frozen by liquid nitrogen, and stored at –80°C. Cryosections (10 μm) adhered to silan-coated coverslides were fixed with 2% paraformaldehyde and permeabilized with 0.2 % Triton X-100 and blocked with 1% BSA plus 30% NGS. The tissue samples were transferred to an inverted wide-field fluorescence microscope (Leica DMi8, ×20 air lens, NA 0.80). The automated cyclic robotic process started with the incubation of the first fluorescently labeled antibody (tag). After a series of washing steps, the fluorescence signals and a corresponding phase contrast image were acquired by a scientific CMOS camera (Orca Fusion, Hamamatsu). The specific signal of the given tag was removed by bleaching of the fluorescent dye followed by recording of post-bleaching fluorescence signals and repetition of the incubation-imaging-bleaching cycle. The appropriate working dilutions, incubation times, and positions within the MELC experiment were validated systematically using conditions suitable to MELC. The series of fluorescence images produced by each tag were aligned with an accuracy of 0.1 pixels using the corresponding phase contrast images. Illumination faults of the images were corrected using flat-field correction. Post-bleaching images were subtracted from the following fluorescence tag images. Section artifacts were excluded as invalid by a manual mask-setting process. For cell segmentation, we used a summed image of the markers CD45 and CD54 and recalled the general model of Cellpose (47) within our MATLAB analysis workflow. The CellProfiler (48) software was used to generate a mask for the FRC region, defined as Gp38^+^ and CD31^–^ cells using the Gp38 and CD31 signals. For each cell, the MFI and the smallest distance to the reference region FRC was calculated. The resulting matrix of intensities and distances were exported into an FCS file and uploaded to the online cytometric analysis platform cytobank.org for multiparametric analysis.

### Visualization of RANKL in MCs ex vivo and in vitro.

Mouse ear skin from Mcpt5-Cre-iDTR mice (Cre^+^ and Cre^–^) was harvested, embedded, frozen, and sectioned. Sections were fixed in 1% PFA/PBS overnight at 4°C in a humidified chamber, followed by permeabilization in methanol/acetone (1:1) for 2 minutes on ice. After washing with PBS, MCs were streptavidin blocked (0.5 μg/mL, 60 min), followed by staining with labeled avidin–Alexa Fluor 488 (50 ng/mL, 60 min, RT). Sections were then blocked for 60 minutes at RT and incubated with anti–RANKL PE primary antibody (1:80) overnight at 4°C. After washing (PBS, 0.1% Tween 20), sections were incubated with goat anti–rat Alexa Fluor 647 secondary antibody (1:500) for 1 hour at RT. Slides were washed, air-dried, and mounted for imaging.

For in vitro experiments, MCs (Cre^+^ and Cre^–^ from Mcpt5-Cre-iDTR mice) were isolated and cultured and then cytospun onto glass slides (1 × 10^5^ cells per slide). Cells were stained using the same protocol as described for the skin sections. Images were obtained with a ×63 objective and *z*-stack spacing of 0.3 μm.

### Dermal skin cytokine and serum S1P quantification.

To enable the detection of cytokines, mice were sensitized and challenged. Then, ear samples were collected at 48 hours, and serum was collected at 2, 4, 6, 12, 24, and 48 hours. Ear skin samples were shock-frozen in liquid nitrogen, suspended in serum-free medium, and then mechanically homogenized using a tissue homogenizer mill with stainless steel or ceramic beads. Cytokines of the ear skin were quantified using a Legendplex assay (BioLegend) according to the manufacturer’s instructions and were normalized to the skin biopsy weight. S1P was quantified using a reverse inhibition ELISA (Biomatik) according to the manufacturer’s instructions.

### Isolation and culturing of LECs.

LECs were isolated from C57B/6J mice. The mice were sacrificed for scientific purposes using CO_2_ inhalation. The ears of each mouse were collected, separated into dermal sheets, and minced into 1 mm^2^ pieces and then enzymatically digested with 198 U/mL DNAse, 0.03125 mg/mL Liberase (Roche), and 0.25 mg/mL hyaluronidase in unsupplemented RPMI, followed by vigorous shaking at 37°C for 1 hour. The cell suspension was filtered through a 100 μm filter, and the cells were pelleted at 300*g* for 5 minutes at 4°C. The supernatant was discarded, and the cells were resuspended in MACs buffer (PBS, 2 mM EDTA, 0.5% w/v BSA) containing 10 μL/mL LYVE-1-biotin, 15 μL/mL CD45–eFluor 450, and 15 μL/mL CD31-AF488 antibodies and incubated for 15 minutes at 4°C. The primary antibodies were washed with MACs buffer, and the cells were pelleted at 300*g* for 5 minutes at 4°C, resuspended in MACs buffer containing 10 μL/mL streptavidin-AF647, and incubated for 30 minutes at 4°C. Following incubation, the cells were washed with MACs buffer, pelleted, resuspended in fresh MACs buffer, and sorted using the BD ARIA III Cell Sorter. From the CD45^–^ cell population, the population that was double-positive for LYVE-1 and CD31 was collected. After sorting, the cells were pelleted and resuspended in high-glucose DMEM (PAN Biotech) supplemented with 20% FCS,100 U/mL penicillin, 100 μg/mL streptomycin, 2 mM l-glutamine, 20 mM HEPES, 1% minimally essential amino acids, 1 mM sodium pyruvate, 50 μM 2-mercaptoethanole, 75 μg/mL endothelial growth factor, 1 U/mL heparin, 10 μg/mL amphotericin B, and 10 μg/mL Fungin (Thermo Fisher Scientific). The cells were seeded in a well on a 24-well plate coated with 2% gelatin and incubated at 37°C in a 5% CO_2_ environment. The media were replaced the day after cell sorting, and then every 2–3 days. Amphotericin B was removed from the culture after 3–4 days and Fungin after 1 week.

After cell sorting, the cells were allowed to grow to 80% confluence before they were subcultured in a larger container. When subcultured, the cells were washed with PBS to remove protein residues and incubated with 0.5% trypsin EDTA at 37°C in a 5% CO_2_ environment until they detached from the plate. The trypsin was neutralized with complete EC medium, and the cells were pelleted at 300*g* for 5 minutes at 4°C. To remove the trypsin and gelatin residues, the supernatant was discarded, and the cells were resuspended in complete EC medium, before being transferred to a fresh container coated with 2% gelatin. The cells were subcultured 6 times before being characterized or used for experiments.

### Software.

For visualization, 2-photon microscopy raw data were processed and reconstructed using Imaris version 10.1 (Bitplane). Analysis of flow cytometric raw data was performed using FlowJo Analysis Software V10 (FlowJo). Graphic data analysis was done with the OriginPro software (OriginLab) and GraphPad Prism 10 (GraphPad Software). Statistical analysis was performed with GraphPad Prism 10.

### Statistics.

Statistical analysis was performed with the Mann-Whitney *U* test when comparing 2 experimental groups or otherwise using 1-way ANOVA. The respective nonparametric tests were used in the case where a non-Gaussian distribution was found. All graphs are depicted as the mean ± SD.

### Study approval.

All procedures were performed in accordance with institutional guidelines for animal welfare and were approved by the Landesverwaltungsamt Sachsen-Anhalt (animal licenses 203.m-42502-2-1416_UniMD and 203.m-42502-2-1732_UniMD). Mice that didn’t undergo treatment were sacrificed for scientific purposes using CO2 inhalation, according to license number IMKI-TWZ-ADU-1-25.

### Data availability.

All data are available in the Supporting Data Values file and are also available upon request.

## Manuscript information

### Author contributions.

KKD and AD conceived and designed the study, analyzed and discussed data, and wrote the manuscript. KKD, WU, AEB, AH, VD, LK, T Schickschneit, CH, LMS, LP, KB, NJN, and JD performed experiments and analyzed data. AJM, SK, T Schüler, SF, and JD provided substantial guidance and expertise during the revision process, including advice on experimental design and critical review of the manuscript’s content. All authors revised the manuscript and approved the final version of the manuscript.

### Conflict of interest.

LP is affiliated with BioDecipher GmbH, a company that develops systems for multiplexed fluorescence microscopy (MELC).

### AI disclosure.

The authors declare that no AI tools were used in the original research for or writing of this article.

### Funding.

This work was funded by the following:

- Deutsche Forschungsgemeinschaft (DFG) (German Research Foundation; project IDs: 361210922/RTG2408/TP4, DU1172/8-1, and DU1172/9-1, to AD).
- European Union Research Fund (EFRE) Sachsen-Anhalt ‘Wissenschaft, Forschung und Innovation’ (ZS/2024/05/186745).
- Health Campus for Inflammation, Immunology and Infectiology of the Medical Campus of the Otto-von-Guericke University (GC-I3).

### Present address.

WU’s present address is: University Clinic for Gastroenterology, Hepatology and Infectiology, University Hospital Magdeburg, Magdeburg, Germany.

## Supplementary Material

Supplemental data

Supporting data values

## Figures and Tables

**Figure 1:**
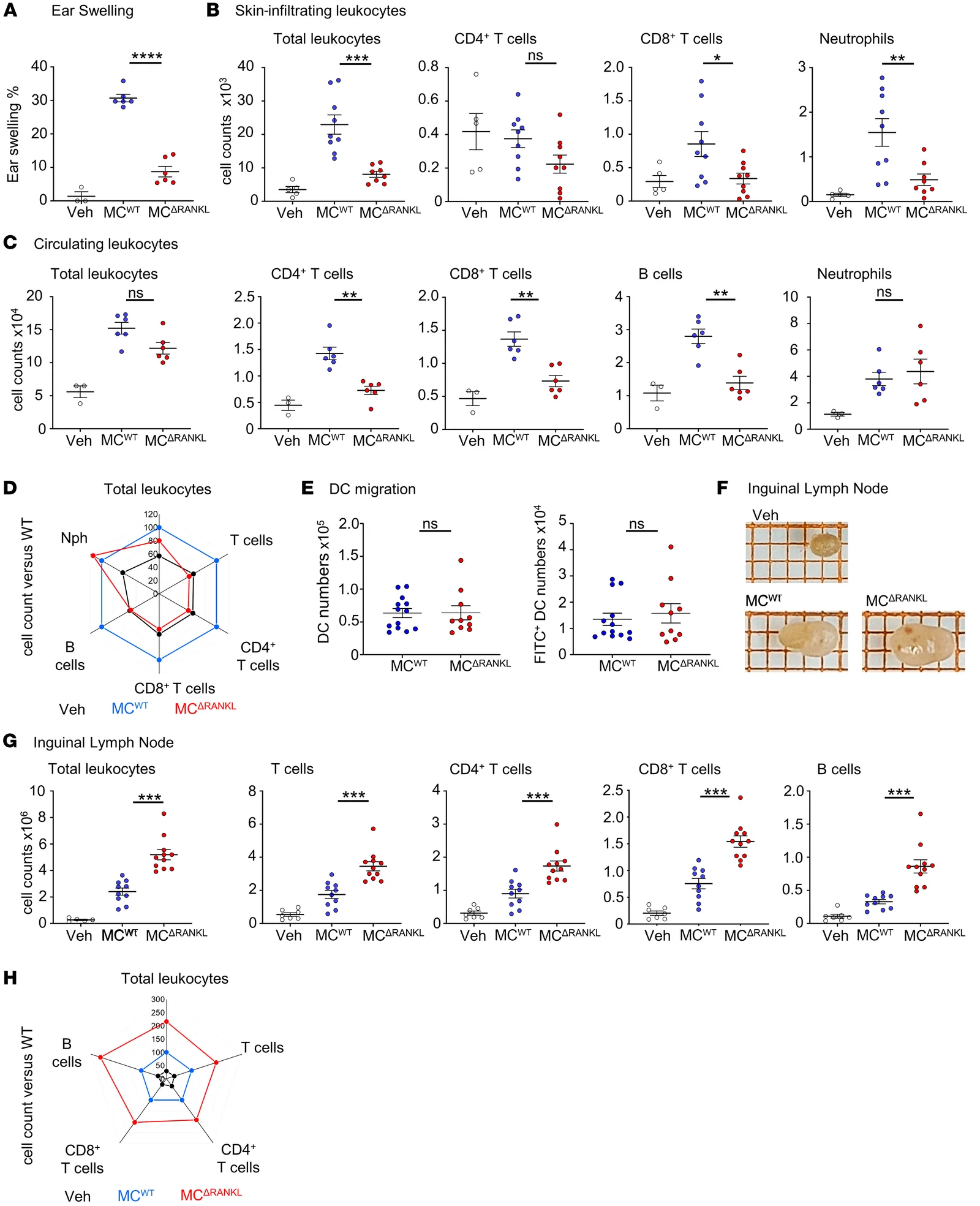
Absence of MC-derived RANKL results in blood lymphopenia, but inguinal LN hyperplasia upon CHS elicitation. Mice were sensitized on the back skin and after 6 days challenged on the ear skin for 24 hours. (**A**) Ear skin swelling response. (**B**) Total numbers of lymphocytes infiltrating the ear skin. (**C**) Blood lymphocyte numbers per 100 μL blood. (**D**) Radar plot showing blood lymphocyte numbers per 100 μL blood. MC^WT^ was set as the baseline. (**E**) MC^ΔRANKL^ or MC^WT^ mice were sensitized by epicutaneous application of 100 μL FITC on the shaved back. Forty-eight hours after sensitization, inguinal LNs were isolated and total numbers of DCs (left) and FITC^+^ DCs that had migrated from the skin (right) were analyzed by flow cytometry. (**F**) Side-by-side comparison of whole inguinal LNs from MC^ΔRANKL^ and MC^WT^ and vehicle-treated mice. (**G**) Inguinal LN lymphocyte numbers. (**H**) Radar plot showing total cell count in inguinal LNs, normalized by setting MC^WT^ at 100%. Data in all graphs are depicted as the mean ± SD. *P < 0.05, **P < 0.01, ***P < 0.001, and ****P < 0.0001, by Mann-Whitney *U* test (**E**) or Kruskal-Wallis test with correction for multiple comparisons (**A**, **B**, **C**, and **G**). Veh, vehicle.

**Figure 2:**
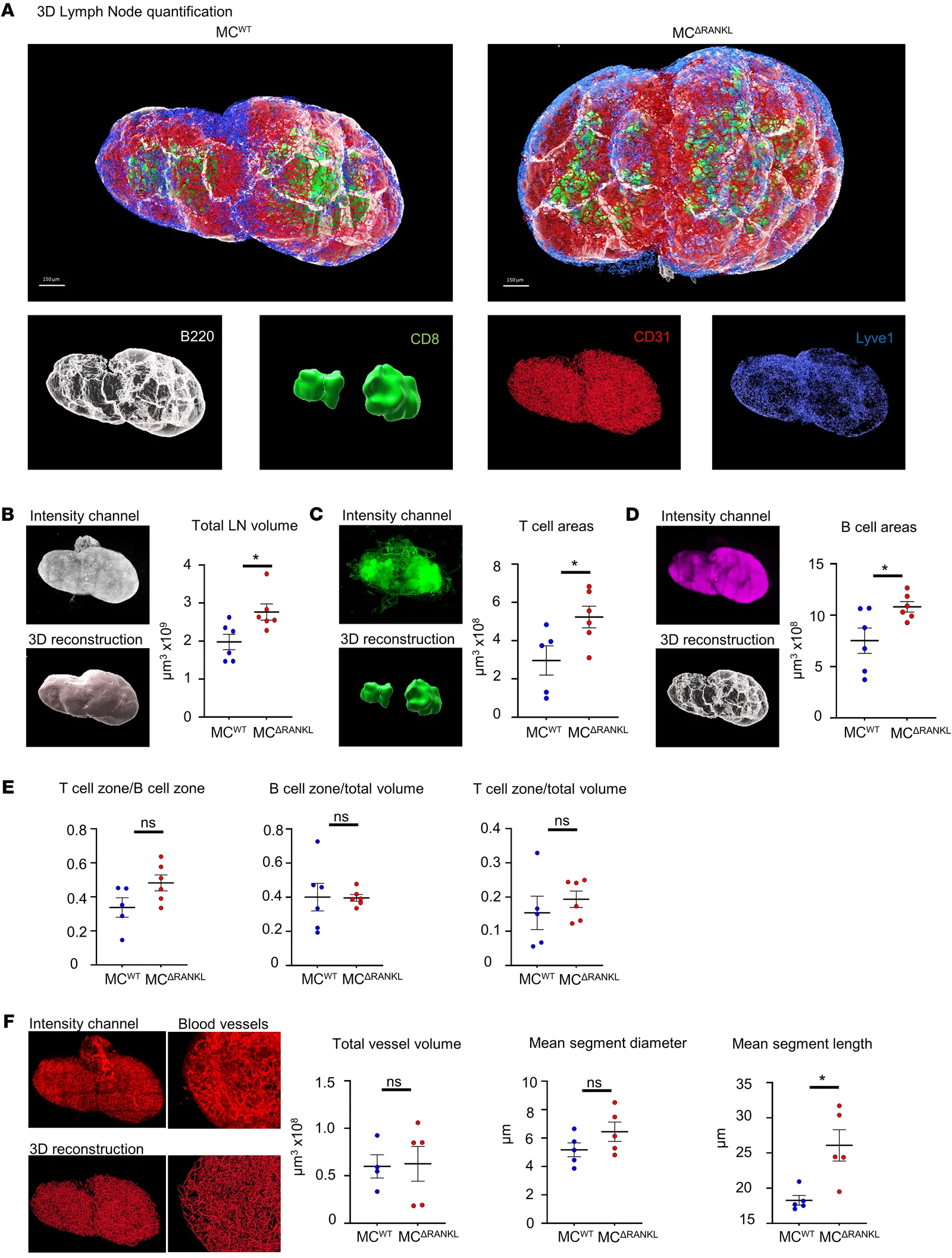
LN hyperplasia in MC^ΔRANKL^ mice is not driven by structural maladaptation. (**A**) Whole-mount staining, 3D imaging, and reconstruction of inguinal LNs from MC^WT^ and MC^ΔRANKL^ mice, 24 hours after hapten challenge, including B cell (B220) and T cell zones (CD8), blood vessels (CD31), and lymphatic vessels (Lyve1). Scale bars: 100 μm. (**B**) Total inguinal LN volume was measured by creating a surface based on all channels with Imaris machine learning and is plotted in the cumulative graph. (**C** and **D**) T cell (**C**) and B cell (**D**) area volume was measured by creating a surface with Imaris and plotted on the cumulative graph. (**E**) B cell and T cell areas are shown as their ratio with each other and with the total volume of the inguinal LN. (**F**) Blood vessels were reconstructed with the Imaris filament tracer. Cumulative graphs of blood vessel volume, segment diameter, and length. The images in **B**, **C**, **D**, and **F** are different depictions of the images in **A**. All graphs are depicted as the mean ± SD. *P < 0.05, by Mann-Whitney *U* test (**B**, **C**, **D**, **E**, and **F**).

**Figure 3:**
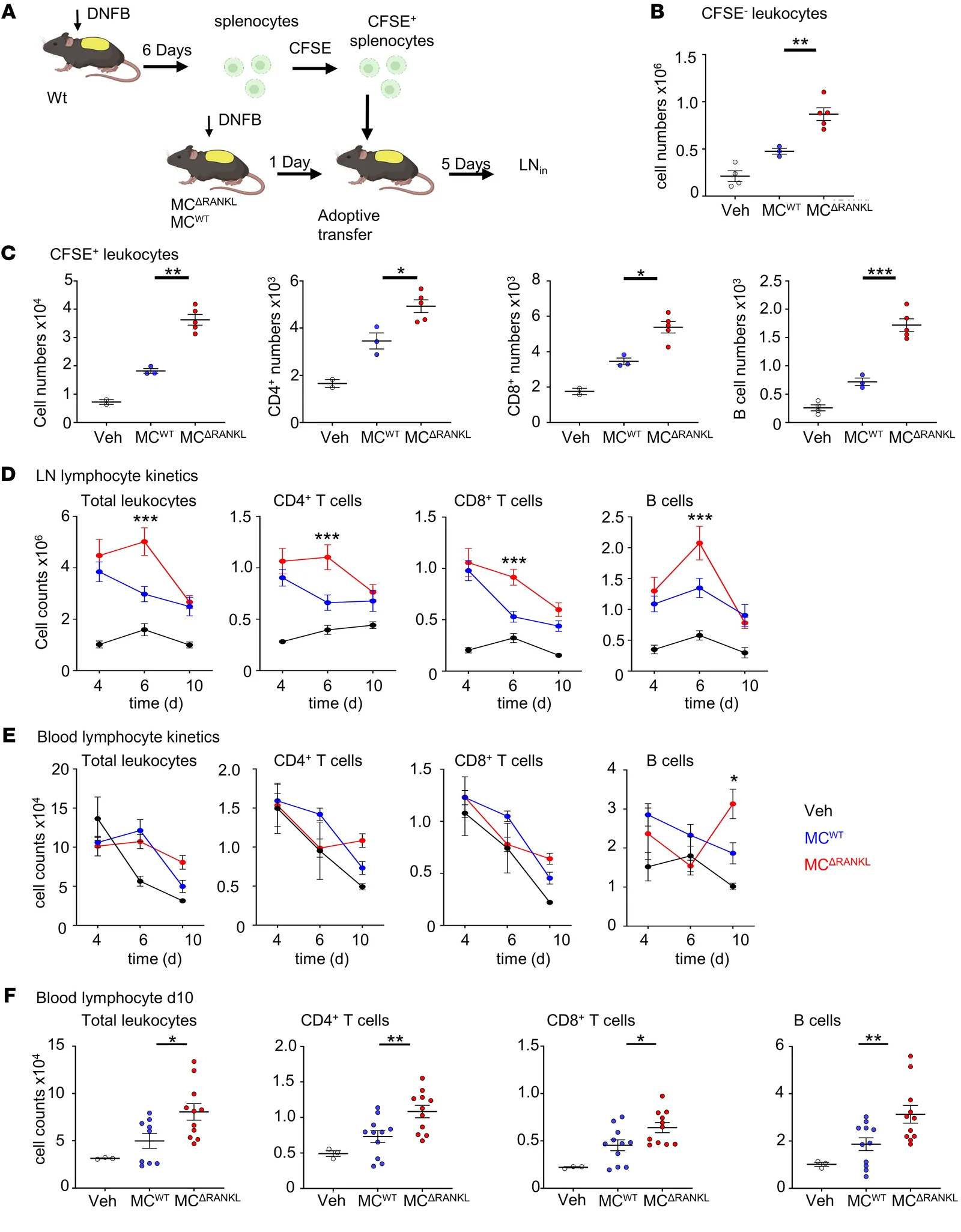
Inguinal LN hyperplasia in MC^ΔRANKL^ mice is transient and driven by tissue effects. (**A**) MC^ΔRANKL^ or MC^WT^ mice as well as WT mice were sensitized on the back skin for 6 days. Lymphocytes were isolated from spleens and LNs of sensitized WT mice, labeled with CFSE, and adoptively transferred into MC^ΔRANKL^ or MC^WT^ mice 1 day after sensitization. Five days later, flow cytometric analysis of LN lymphocytes was performed. (**B**) Total numbers of lymphocytes in the inguinal LN. (**C**) Numbers of CFSE^+^-labeled lymphocyte subsets in inguinal LNs of recipient mice. (**D**) Mice were sensitized on the back skin with DNFB, and total leukocyte numbers were evaluated by flow cytometry on days 4, 6, and 10. Kinetics assay of inguinal LN_i_ lymphocyte numbers at days 4, 6, and 10 after sensitization. (**E**) Kinetics assay of blood lymphocyte numbers, per 100 μL blood, at days 4, 6, and 10 after sensitization. (**F**) Blood lymphocyte numbers per 100 μL blood at day 10 after sensitization. All graphs are depicted as the mean ± SD. *P < 0.05, **P < 0.01, and ***P < 0.001, by Kruskal-Wallis test (**B**, **C**, and **F**) or 2-way ANOVA (**D** and **E**), both with correction for multiple comparisons.

**Figure 4:**
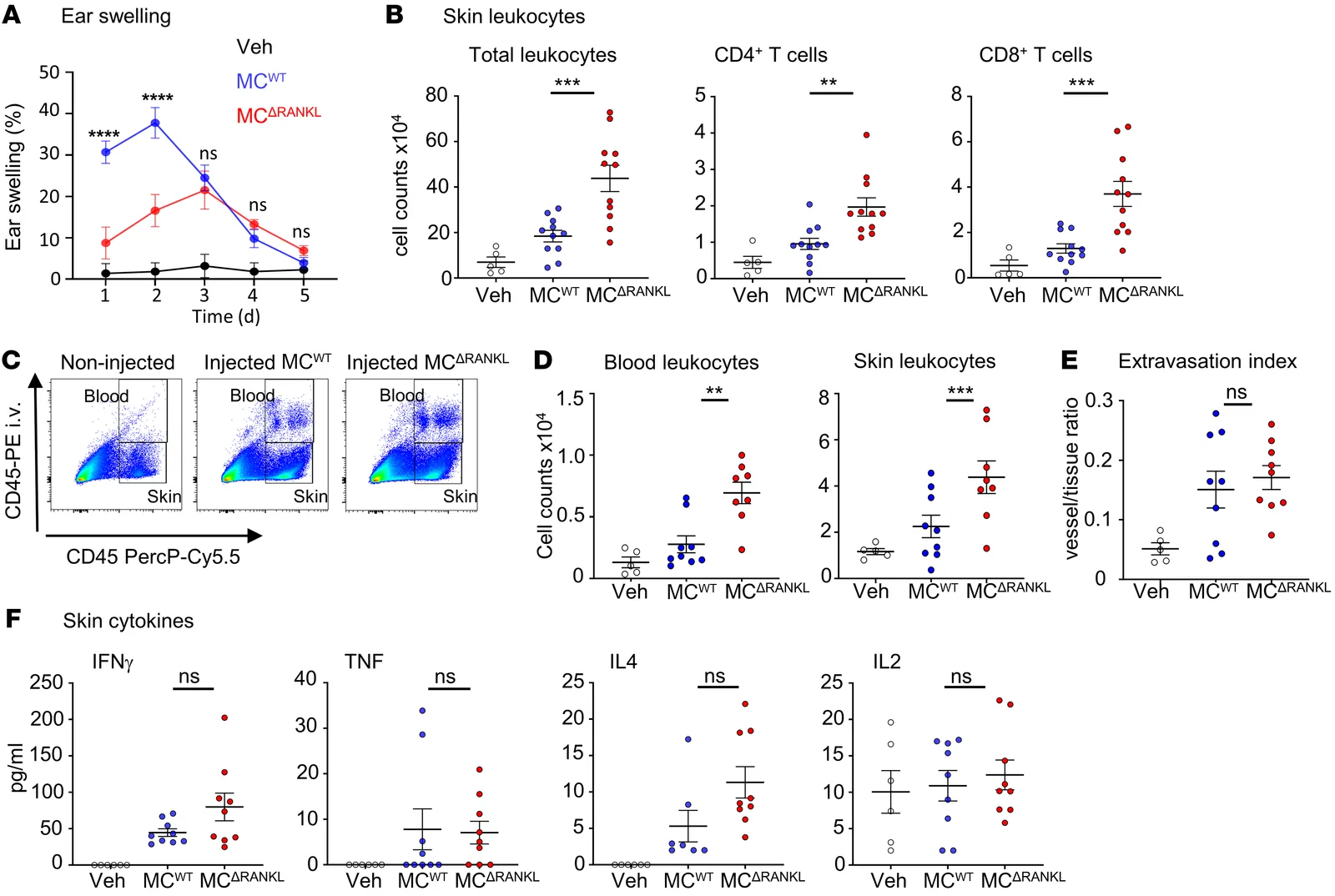
Delayed onset of leukocyte skin infiltration leads to reduced inflammatory symptoms. Mice were sensitized on the back skin and challenged 6 days later on the ear skin for 48 hours. (**A**) Kinetics of the ear swelling response up to day 5 after DNFB challenge. (**B**) Total numbers of lymphocytes infiltrating the ear skin 48 hours after challenge. (**C**) Example FACS plot showing the gating strategy for separating vessel lymphocytes to tissue lymphocytes. MC^ΔRANKL^ or MC^WT^ were sensitized and challenged 6 days later on the ear skin. CD45-PE antibody was injected i.v. 48 hours after challenge in order to stain all circulating leukocytes. After ear skin digestion, circulating PE-labeled leukocytes were quantified in relation to total leukocytes, stained ex vivo with CD45-PerCP-Cy5.5 to separate lymphocytes in the tissue from the cells in the vessels. (**D**) Graphs showing lymphocyte numbers in the blood vessels and ear skin of the ears. (**E**) Cumulative graph showing extravasation as a normalized fraction of cells in the vessels to cells in the tissue. (**F**) Inflammatory cytokine concentrations in the ear skin were measured 48 hours after challenge. All graphs are depicted as the mean ± SD. **P < 0.01, ***P < 0.001, and ****P < 0.0001, by Kruskal-Wallis test (**B**, **D**, **E**, and **F**) or 2-way ANOVA (**A**), both with correction for multiple comparisons.

**Figure 5:**
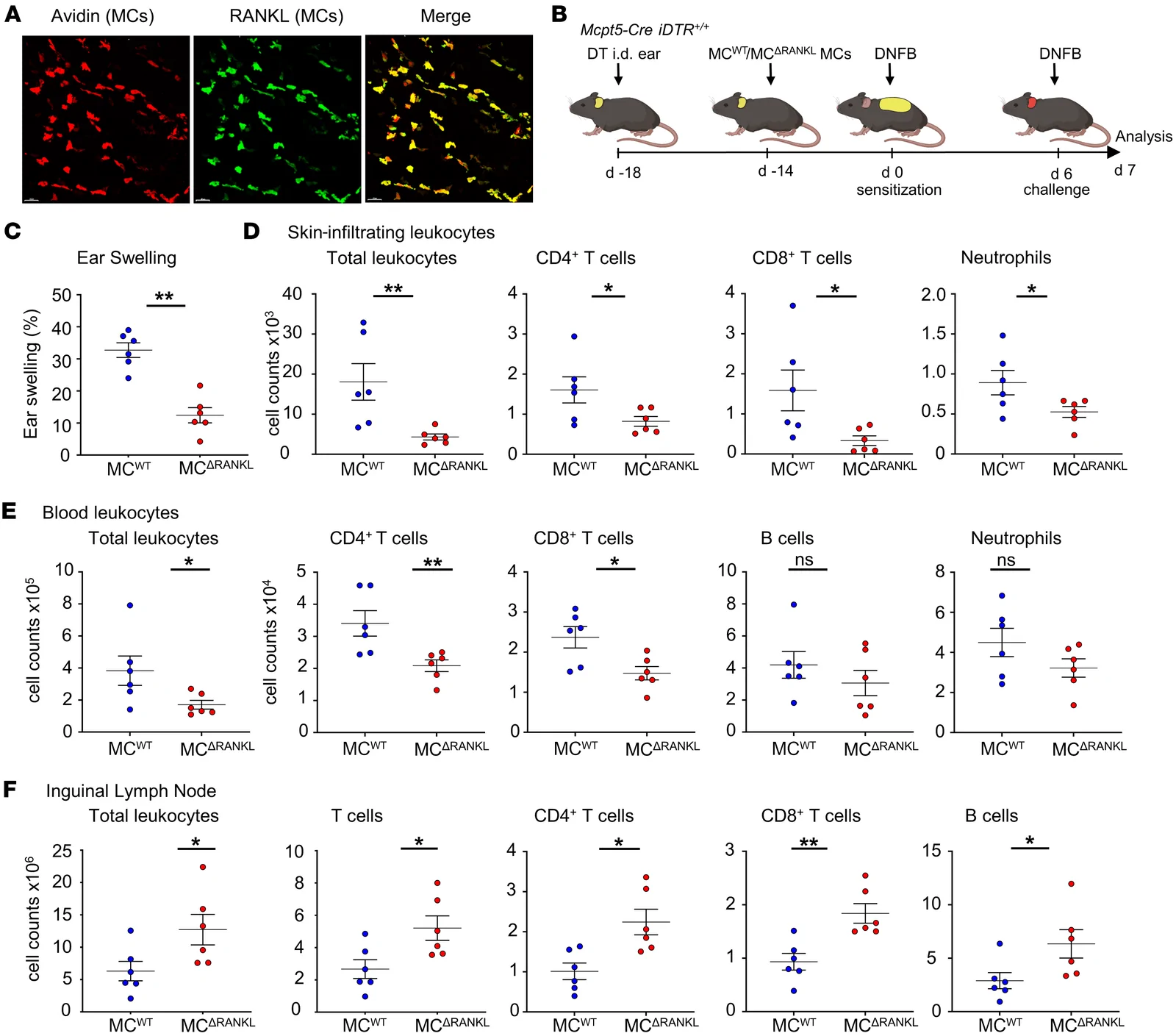
RANKL release by peripheral skin MCs is crucial for lymphocyte egress from distant inguinal LNs. (**A**) Confocal microscopy of whole-mount dermal sheets showing RANKL expression by skin MCs in the steady state. Scale bars: 10µm. (**B**) MCs were depleted only locally in the ear skin by i.d. DT injection in Mcpt5-Cre iDTR mice. Subsequently, ear skin MCs were reconstituted with peritoneally cultured MCs either from MC^ΔRANKL^ or MC^WT^ mice, followed by sensitization and challenge with DNFB. After 24 hours, lymphocyte numbers were validated by flow cytometry. d, day. (**C**) Ear skin swelling response. (**D**) Total number of lymphocytes infiltrating the ear skin 24 hours after challenge. (**E**) Blood lymphocyte numbers per 100 μL blood as quantified by flow cytometry. (**F**) Total number of lymphocytes in inguinal LNs 24 hours after challenge. Data in all graphs are depicted as the mean ± SD. *P < 0.05 and **P < 0.01, by Mann-Whitney *U* test (**C**, **D**, **E**, and **F**).

**Figure 6:**
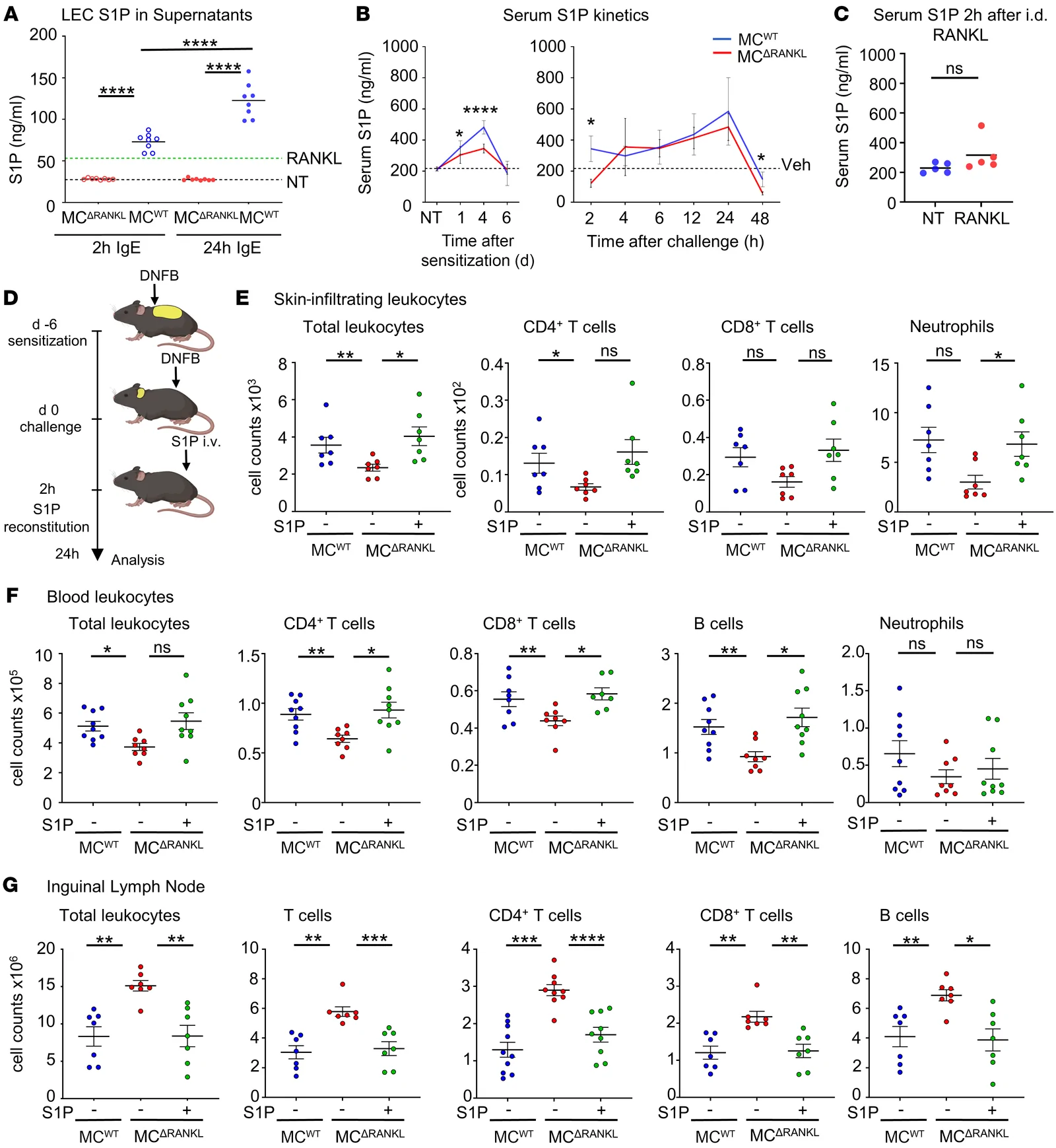
S1P administration rescues LN lymphocyte retention in MC^ΔRANKL^ mice. (**A**) LECs were sorted from ear skin and expanded in vitro and then treated with PCMC supernatants that were either activated for 2 hours or 24 hours via the FceR in vitro (anti–DNP-IgE for 24 hours followed by DNP BSA). LECs were also treated with recombinant RANKL (green line) or were untreated (black line). S1P expression was measured in the supernatants. (**B**) MC^ΔRANKL^ or MC^WT^ were DNFB sensitized and challenged after 6 days, and a kinetic assay of S1P was performed during sensitization and up until 48 hours after challenge. (**C**) Recombinant RANKL was injected i.d. into the ear and 2 hours after serum S1P was measured. (**D**) Two hours after challenge, 500 ng S1P (+) or PBS only as a control (–) was i.v. injected. Flow cytometric measurements of skin, blood, and LN lymphocytes were performed 24 hours after challenge. (**E**) Total numbers of lymphocytes infiltrating the ear skin. (**F**) Blood lymphocyte numbers per 100 μL blood. (**G**) Total numbers of lymphocytes in inguinal LNs. All graphs are depicted as the mean ± SD. *P < 0.05, **P < 0.01, ***P < 0.001, and ****P < 0.0001, by Kruskal-Wallis test (**A**, **E**, **F**, **G**), or 2-way ANOVA (**B**), both with correction for multiple comparisons, or by Mann-Whitney *U* test (**C**).

## Version 1 09/24/2026

In-Press Preview

## Version 2 11/16/2026

Electronic publication
