# Supplementary material for: Mast cells initiate lymphocyte egress from distant lymph nodes upon skin inflammation via RANKL/sphingosine-1-phosphate axis
Source: J Clin Invest. 2026 Sep 24;136(22):e199237. doi: 10.1172/JCI199237 (PMC13767624; doi:10.1172/JCI199237)
Supplement: Supplementary file 1 [file jci-136-199237-s069.pdf]

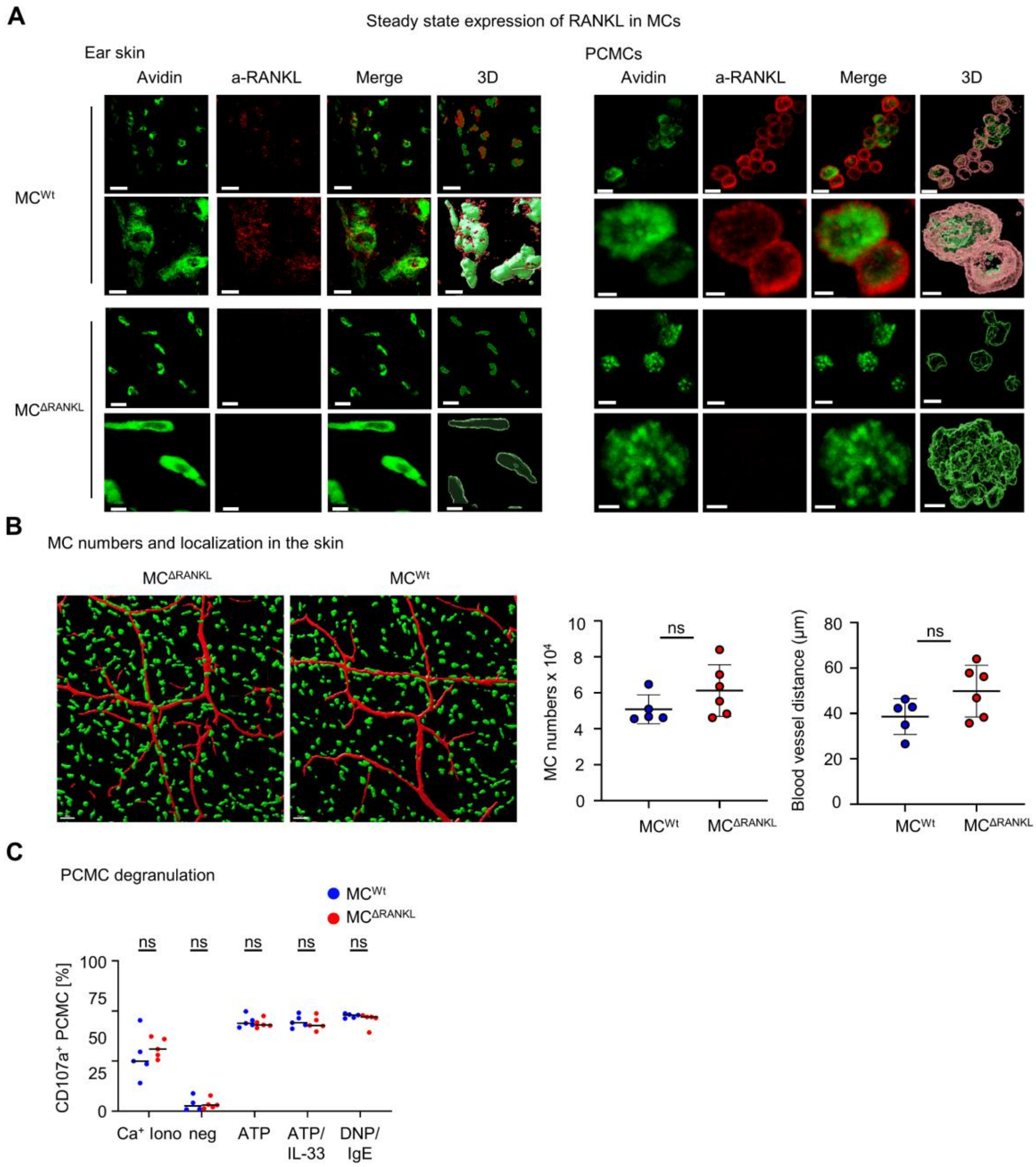

Supplemental Figure 1: Absence of MC-derived RANKL does not affect MC degranulation and skin localization. (**A**) RANKL expression in $MC^{\Delta RANKL}$ and MC^{Wt} mice in whole mount dermal sheets (left panel) and peritoneal cultured mast cells (PCMCs) (Right panel) (**B**) Whole mount ear dermal sheet microscopy of $MC^{\Delta RANKL}$ and MC^{Wt} mice. MC numbers and distance to the blood vessels were quantified with Imaris software. (**C**) PCMCs were cultured and degranulation was induced with different stimuli All graphs are depicted as mean and SD. **** $p < 0.0001$; *** $p < 0.001$; ** $p < 0.01$; * $p < 0.05$. The following statistical tests were used, Mann-Whitney test (B,), Kruskal-Wallis test (C) with correction for multiple comparisons.

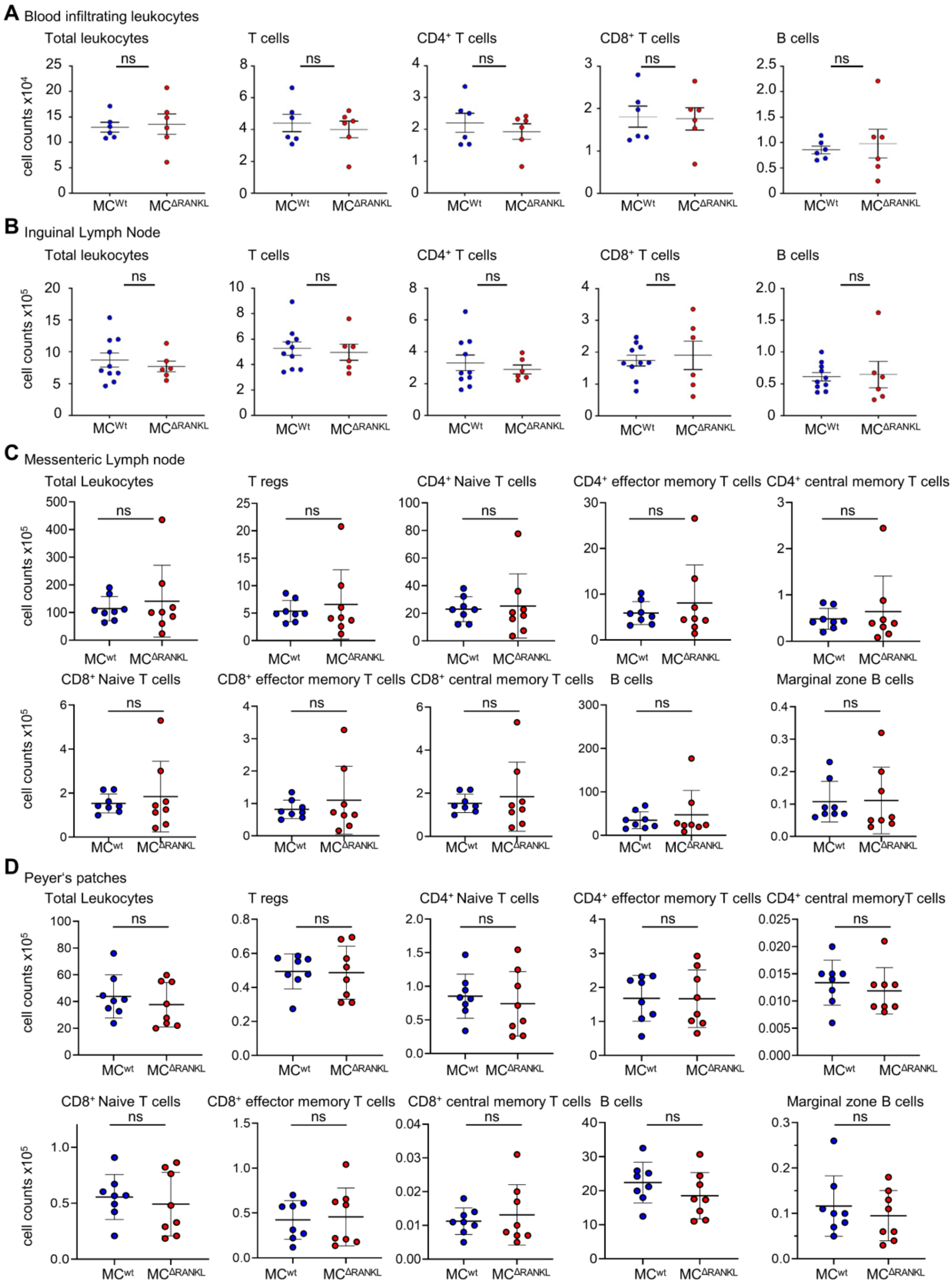

Supplemental Figure 2: Absence of MC-derived RANKL does not affect steady state leukocyte populations. Blood and LN lymphocyte numbers under physiologic conditions were analysed by flow cytometry. (**A**) Blood lymphocyte numbers per 100µl of blood. (**B**) Total

numbers of lymphocytes in LN_{in}. (**C**) Total numbers of lymphocytes in mesenteric LNs. (**D**)Total lymphocyte numbers in Peyer's Patches. All graphs are depicted as mean and SD. **** $p < 0.0001$; *** $p < 0.001$; ** $p < 0.01$; * $p < 0.05$. Mann-Whitney test was used for statistical significance.

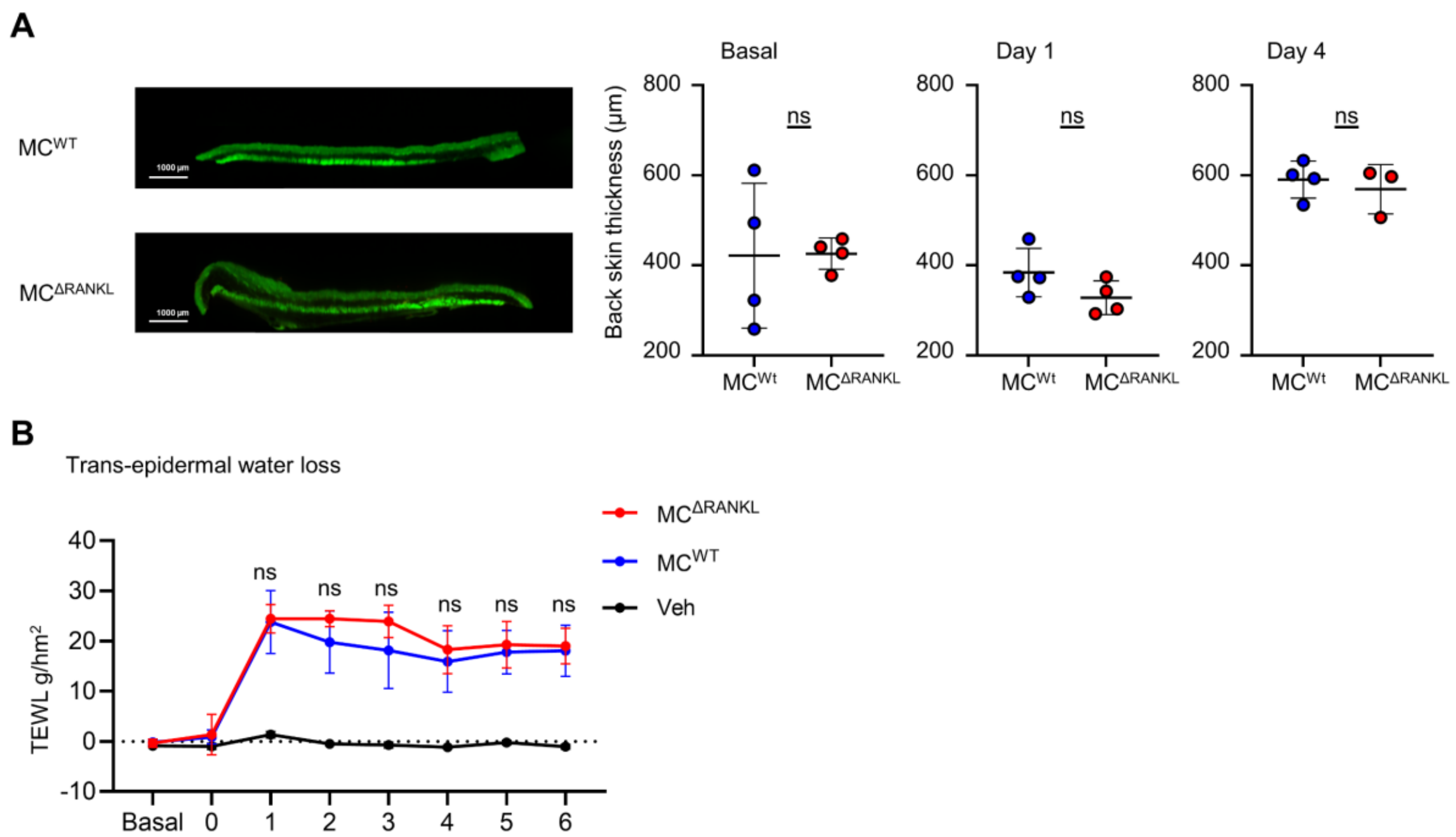

Supplemental Figure 3: Absence of MC-derived RANKL does not affect skin thickness or trans-epidermal water loss during sensitization with DNFB. (**A**) Dermal thickness of back skin in the steady state and on days 1 and 4 after sensitization with DNFB, as visualized by light sheet microscopy. (**B**) Trans-epidermal water loss kinetic of shaved back skin during sensitization with DNFB. All graphs are depicted as mean and SD. **** $p < 0.0001$; *** $p < 0.001$; ** $p < 0.01$; * $p < 0.05$. The following statistical tests were used, Mann-Whitney test (A), 2-way ANOVA (B) with correction for multiple comparisons.

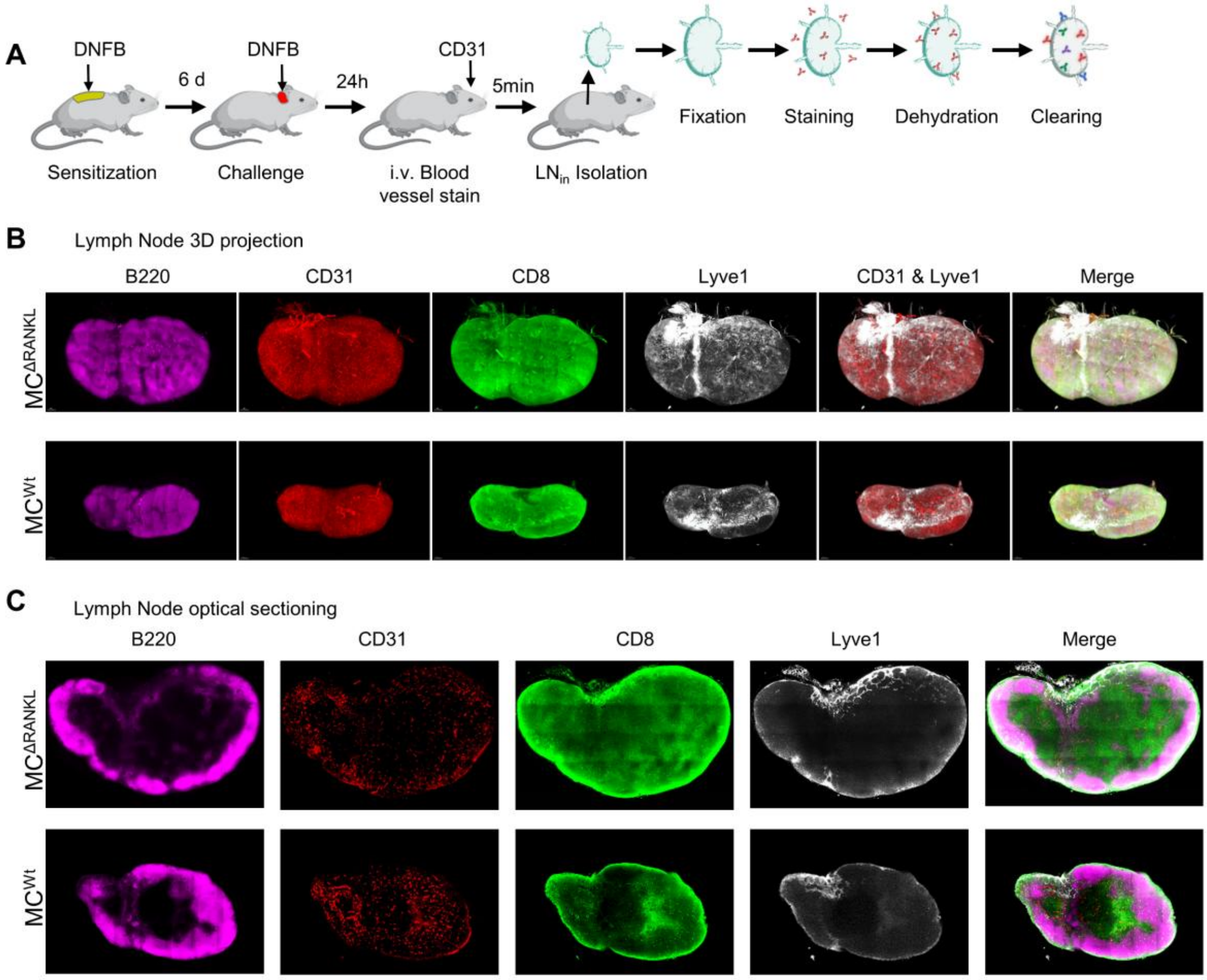

Supplemental Figure 4: LN_{in} hyperplasia in $MC^{\Delta RANKL}$ mice was investigated by advanced whole mount 3D microscopy. (**A**) Mice were sensitized on the back skin and after 6 days challenged on the ear skin. 24h after challenge LN_{in} were collected, stained with antibodies and cleared. LNs were stained with B220 for the B cell compartments, i.v. CD31 for the blood vessels, CD8 for the T cell compartments and Lyve1 for the lymphatic vessels and visualized with intravital two photon microscopy. (**B**) 3D lymph node projection. (**C**) Optical sections of LN_{in} were taken from the midpoint of the imaging plane.

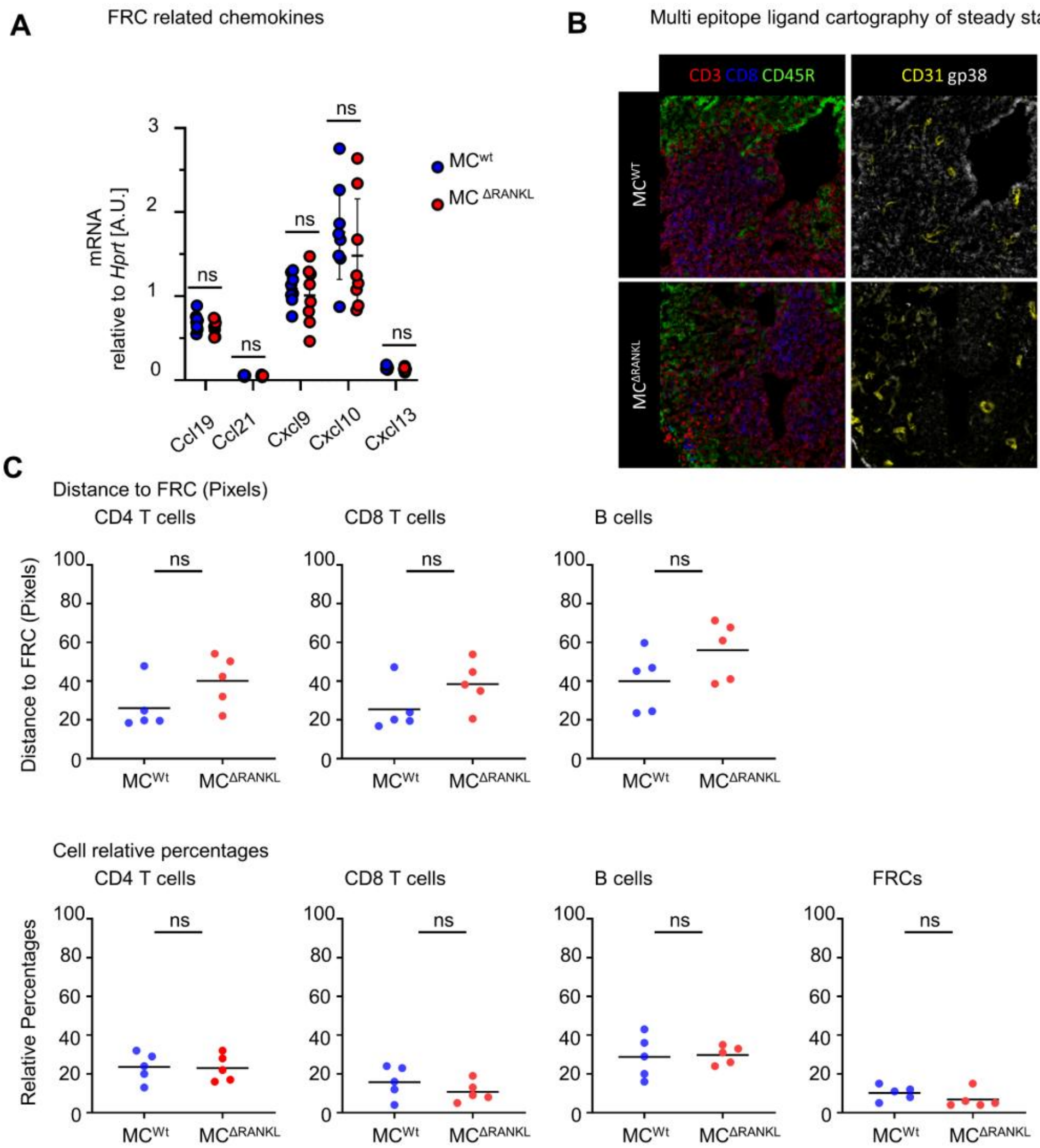

Supplemental Figure 5: Absence of MC-derived RANKL does not affect LN fibroblastic reticular cell localization and function in the steady state. (**A**) FRC relevant chemokine mRNA expression from whole inguinal LNs of MC$^{\Delta RANKL}$ and MC^{Wt} mice. (**B**) Multi epitope ligand cartography was performed to identify FRCs, CD4 T cells, CD8 T cells and B cells in inguinal LN sections and (**C**) their distances from FRCs were calculated. The following statistical tests were used, Mann-Whitney test (C), Kruskal-Wallis test with correction for multiple comparisons (A).

REAGENT or RESOURCE	SOURCE	IDENTIFIER
Antibodies		
Hamster monoclonal anti-mouse CD3 (145-2C11) PE-Cy7	Biolegend	Cat# 100320; RRID AB_312685
Hamster monoclonal anti-mouse CD3 (145-2C11) PE	eBiosciences	Cat# 12-0033-82; RRID AB_842786
Rat monoclonal anti-mouse CD4 (GK1.5) Percp-efluor-710	eBiosciences	Cat# 46-0041-82; RRID AB_11150050
Rat monoclonal anti-mouse CD4 (GK1.5) AF488	eBiosciences	Cat# 53-0041-82; RRID AB_469892
Rat monoclonal anti-mouse CD8 (53-6.7) BV510	Biolegend	Cat# 100752; RRID AB_2563057
Rat monoclonal anti-mouse CD8 (53-6.7) AF488	Biolegend	Cat# 100723; RRID AB_389304
Rat monoclonal anti-mouse CD25 (PC61) BV421	Biolegend	Cat# 102043; RRID AB_2562611
Rat monoclonal anti-mouse CD44 (IM7) PE	Biolegend	Cat# 103008; RRID AB_312959
Rat monoclonal anti-mouse B220 (MAR-1) AF647	BD Pharmingen	Cat# 557683; RRID AB_396793
Rat monoclonal anti-mouse B220 (MAR-1) FITC	Biolegend	Cat# 103206; RRID AB_312991
Rat monoclonal anti-mouse Ly6G (1A8) PE-Cy7	Biolegend	Cat# 127 618; RRID AB_1877261
Rat monoclonal anti-mouse Ly6G (1A8) APC	Biolegend	Cat# 127614; RRID AB_2227348
Rat monoclonal anti-mouse F4/80 (BM8) APC-efluor780	eBiosciences	Cat# 47-4801-82; RRID AB_2735036
Rat monoclonal anti-mouse CD11b (M1/70) Efluor-450	eBiosciences	Cat# 48-0112-82; RRID AB_1582237
Rat monoclonal anti-mouse Lyve1 (ALY7) Biotin	eBiosciences	Cat# 13-0443-82; RRID AB_1582237
Rat monoclonal anti-mouse CD31 (390)	Biolegend	Cat# 102432; RRID AB_2617017
Rat monoclonal anti-mouse CD31 (MEC13.3)	BD	Cat# 550274 RRID AB_393571
Rat monoclonal anti-mouse CD45 PE	Biolegend	Cat# 103106; RRID AB_312971
Rat monoclonal anti-mouse CD45 efluor-450	eBiosciences	Cat# 48-0451-82 RRID AB_312971
Rat monoclonal anti-mouse CD45 PercP-Cy5.5	Biolegend	Cat# 103132; RRID AB_893340
Goat polyclonal anti-rabbit AF488	ThermoFisher	Cat# A-11006
Goat polyclonal anti-rat AF647	Jackson ImmunoResearch	Cat# 112-605-003
Rabbit anti-mouse polyclonal RANKL (CD254)	antibodies online	Cat# ABIN668556
Rat anti-mouse monoclonal RANKL (CD254)	Biolegend	Cat# 112-605-003 RRID AB_2303587
Mouse anti-mouse monoclonal αSMA (1A4) eFluor660	eBioscience	Cat# 50-9760-82 RRID AB_2574362

Rat monoclonal anti-mouse endomucin (eBioV.7C7) eFluor660	eBioscience	Cat# 50-5851-80 RRID AB_11220069
Anti-rat polyclonal DyLight 800	invitrogen	Cat# SA5-10032 RRID AB_2556612
Mouse monoclonal anti-mouse Dinitrophenyl (DNP) IgE	Sigma-Aldrich	Cat# D8406; AB_259249
Hamster monoclonal anti-mouse FcεRI (MAR-1) AF647	Biolegend	Cat# 134309; AB_1626097
Rat monoclonal anti-mouse CD117 (ACK2) BV421	Biolegend	Cat# 135124; AB_2562237
Rat monoclonal anti-mouse CD107a (1D4B) PE	eBiosciences	Cat# 12-1071-82; AB_657554
Bacterial and Virus Strains		
n/a		
Biological Samples		
n/a		
Chemicals, Peptides, and Recombinant Proteins		
DNFB (1-fluoro-2,4-dinitrobenzene)	Sigma-Aldrich	Cat# D1529-25ML
Hyaluronidase	Sigma-Aldrich	Cat# H3506
Liberase™	Roche	Cat# 5401119001
DNase I	Roche	Cat# 10104159001
Olive oil	Sigma-Aldrich	Cat# O1514-100ML
Aceton	Fisher Scientific/J.T.Baker	Cat# 8002
Streptavidin BV421	Biolegend	Cat# 405226
Sphingosine 1 Phosphate	Sigma Aldrich	Cat# 73914-1MG
Thioglycerol	Sigma-Aldrich	Cat# 88640-100ML
Ethyl Cinnamate	Sigma-Aldrich	Cat# 199179-92-5
CellTrace™ CFSE Cell Proliferation Kit	Thermo Fisher	Cat# C34554
Sytox DeepRed nucleus dye	invitrogen	Cat# S34859
AF633 hydrazide	life technologies	Cat #A30634
Streptavidin	Jackson ImmunoResearch	Cat# 016-000-113
N,N,N′,N′-Tetrakis(2-hydroxypropyl)ethylendiamin (Quadrol)	Tokyo Chemical Industry	Cat #T0781
DAPI	Sigma-Aldrich	Cat# 32670-5MG-F
Avidin TexasRed	MolecularProbes	Cat# A820
Avidin AF488	MolecularProbes	Cat# A21370
Hair removal cream	Veet	Cat# 3018119
Vectashield antifade mounting medium	Biozol	Cat# VEC-H-1000
2,4-Dinitrophenylated (DNP)-BSA (Albumin from Bovine Serum)	Thermo Fisher	Cat# A23018
Calcium Ionophore A23187	Sigma Aldrich	Cat# C7522
Adenosine 5′-triphosphate disodium salt trihydrate (ATP)	Roche	Cat# 10519979001
Mouse IL-33 Recombinant Protein	PeproTech	Cat# 210-33
Zombie NIR™ Fixable Viability Kit	Biolegend	Cat# 423106
Critical Commercial Assays		

Legendplex mix and match subpanel	Biolegend	Cat# CLT100520
Sphingosine-1-Phosphate (S1P) ELISA Kit	Biomatik	Cat# EKU11440
Deposited Data		
n/a		
Experimental Models: Cell Lines		
n/a		
Experimental Models: Organisms/Strains		
Mcpt5-Cre x iDTR (*Mus musculus*) (Tg(Cma1-cre)ARoer x Gt(ROSA)26Sor$^{tm1(HBEGF)Awai}$)	Central Animal Facility, OvGU Magdeburg	
Mcpt5-Cre x RANKL$^{Fl/Fl}$ (*Mus musculus*) Tg(Cma1-cre)ARoer x B6.129-Tnfsf11$^{tm1.1Caob}$/J	Central Animal Facility, OvGU Magdeburg	
Software and Algorithms		
Imaris (for Windows) Version 9.5	Oxford Instruments, Zurich, Switzerland	N/A
FlowJo^{TM} Software (for Windows) Version 10	FlowJo Inc.	N/A
GraphPad Prism (for Windows) Version 9.02	Graphpad Software	N/A
ORIGIN-Pro	OriginLab Corporation	N/A

Supplemental table 1: Catalogue of all reagents, mouse models and software used in this paper.
